# Cyclic di-GMP is Essential for the Survival of the Lyme Disease Spirochete in Ticks

**DOI:** 10.1371/journal.ppat.1002133

**Published:** 2011-06-30

**Authors:** Ming He, Zhiming Ouyang, Bryan Troxell, Haijun Xu, Akira Moh, Joseph Piesman, Michael V. Norgard, Mark Gomelsky, X. Frank Yang

**Affiliations:** 1 Department of Microbiology and Immunology, Indiana University School of Medicine, Indianapolis, Indiana, United States of America; 2 Department of Microbiology, University of Texas Southwestern Medical Center, Dallas, Texas, Unites States of America; 3 Institute of Insect Science, Zhejiang University, Hangzhou, China; 4 Division of Vector-Borne Diseases, National Center for Emerging and Zoonotic Infectious Diseases, Centers for Disease Control and Prevention, Fort Collins, Colorado, United States of America; 5 Department of Molecular Biology, University of Wyoming, Laramie, Wyoming, United States of America; Medical College of Wisconsin, United States of America

## Abstract

Cyclic dimeric GMP (c-di-GMP) is a bacterial second messenger that modulates many biological processes. Although its role in bacterial pathogenesis during mammalian infection has been documented, the role of c-di-GMP in a pathogen's life cycle within a vector host is less understood. The enzootic cycle of the Lyme disease pathogen *Borrelia burgdorferi* involves both a mammalian host and an *Ixodes* tick vector. The *B. burgdorferi* genome encodes a single copy of the diguanylate cyclase gene (*rrp1*), which is responsible for c-di-GMP synthesis. To determine the role of c-di-GMP in the life cycle of *B. burgdorferi*, an Rrp1-deficient *B. burgdorferi* strain was generated. The *rrp1* mutant remains infectious in the mammalian host but cannot survive in the tick vector. Microarray analyses revealed that expression of a four-gene operon involved in glycerol transport and metabolism, *bb0240-bb0243*, was significantly downregulated by abrogation of Rrp1. *In vitro*, the *rrp1* mutant is impaired in growth in the media containing glycerol as the carbon source (BSK-glycerol). To determine the contribution of the glycerol metabolic pathway to the *rrp1* mutant phenotype, a *glp* mutant, in which the entire *bb0240-bb0243* operon is not expressed, was generated. Similar to the *rrp1* mutant, the *glp* mutant has a growth defect in BSK-glycerol medium. *In vivo*, the *glp* mutant is also infectious in mice but has reduced survival in ticks. Constitutive expression of the *bb0240-bb0243* operon in the *rrp1* mutant fully rescues the growth defect in BSK-glycerol medium and partially restores survival of the *rrp1* mutant in ticks. Thus, c-di-GMP appears to govern a catabolic switch in *B. burgdorferi* and plays a vital role in the tick part of the spirochetal enzootic cycle. This work provides the first evidence that c-di-GMP is essential for a pathogen's survival in its vector host.

## Author Summary

The Lyme disease pathogen *Borrelia burgdorferi* has two sets of two-component systems, Hk1-Rrp1 and Hk2-Rrp2. The Hk2-Rrp2 signaling system has been shown to modulate differential expression of numerous surface lipoprotein genes and to play an essential role in spirochete transformation from a tick colonizer to a mammalian host-adapted state. In this study, we show that Rrp1, the only diguanylate cyclase in *B. burgdorferi*, is not required for mammalian infection but is essential for spirochete survival in the tick vector. We identify over 39 genes whose expression is influenced by this c-di-GMP signaling system. We further demonstrate that one set of the Rrp1-dependent genes, the *glp* operon for glycerol transport and metabolism, plays an important role in the spirochete adaptation to tick environment and partially accounts for the essentiality of c-di-GMP for *B. burgdorferi* survival in ticks.

## Introduction

Bis-(3′-5′)-cyclic dimeric guanosine monophosphate (c-di-GMP), discovered by Benziman and colleagues in the mid-80s [1], is now widely recognized as a ubiquitous second messenger that modulates many aspects of biological processes in bacteria (for reviews, see [2], [3], [4]). C-di-GMP is synthesized by diguanylate cyclases (DGCs), a group of GGDEF domain-containing proteins, and is broken down by phosphodiesterases (PDEs) that contain a conserved EAL or HD-GYP domain [5], [6], [7], [8], [9], [10], [11]. GGDEF, EAL and HD-GYP domains are among the most abundant domains encoded in bacterial genomes [5], [12]. Numerous studies on c-di-GMP signaling pathways in the *Proteobacteria* revealed that c-di-GMP controls the transition between planktonic and biofilm lifestyles by stimulating the biosynthesis of adhesins and exopolysaccharide matrix substances in biofilms while inhibiting various forms of motility [13], [14], [15], [16], [17], [18], [19]. Several classes of c-di-GMP receptor/effector proteins have been identified [20]. Despite tremendous progress, the role of c-di-GMP in bacterial pathogenesis and the mechanisms of action of c-di-GMP remain poorly understood [4], [21], [22]. Further, very little is known about the function of c-di-GMP beyond *Proteobacteria*.

*Borrelia burgdorferi* is a spirochete that causes Lyme disease, the most prevalent vector-borne infection in the United States [23]. As an obligate pathogen, *B. burgdorferi* has a reduced genome that contains a limited number of genes that are known to be involved in signal transduction and gene regulation [24], [25]. For instance, the genome only has two sets of two-component signal transduction systems: Hk1-Rrp1 (BB0420-BB0419) and Hk2-Rrp2 (BB0764-BB0763), in addition to the chemotaxis CheA-CheY system. On the other hand, the enzootic life cycle of *B. burgdorferi* is complex. It involves two markedly different hosts, an arthropod vector and a small mammal. This unique lifestyle requires *B. burgdorferi* to utilize its limited signaling capabilities for adapting to dramatic changes in host environments during its natural cycle. In this regard, the Hk2-Rrp2 two-component signaling pathway has been shown to modulate differential expression of numerous surface lipoprotein genes and plays an essential role for spirochetal transmission and mammalian infection [26], [27], [28], [29], [30].

Little is known about the function of the second two-component system present in *B. burgdorferi*, Hk1-Rrp1. The response regulator Rrp1 contains an N-terminal response regulator receiver domain and a C-terminal GGDEF domain [8]. Ryjenkov *et al*. demonstrated that recombinant Rrp1 has DGC activity that strictly depends on the phosphorylation status of Rrp1 [8]. The complete enzootic cycle of *B. burgdorferi* and the pathogenesis of the disease can be largely reproduced in the laboratory [31]. Rrp1 is the only GGDEF-domain protein in *B. burgdorferi*, making this organism attractive for uncovering the role of c-di-GMP-mediated signaling in bacterial pathogenesis [5], [8].

Two recent studies have shed light on the potential role that c-di-GMP plays in the life cycle of *B. burgdorferi*. Rogers *et al*. showed that *rrp1* is significantly upregulated upon tick feeding [32]. They also generated an *rrp1* mutant in the non-infectious clone B31 5A13. The mutant showed altered expression of more than 140 genes (8% of the genome) whose functions covered almost all functional categories, including cell envelope biosynthesis, transport, metabolism, chemotaxis, and flagellar biosynthesis [32]. The *rrp1* mutant also showed reduced growth at room temperature and increased serum sensitivity [32]. Another study focused on BB0363, the only EAL-domain protein encoded in the *B. burgdorferi* genome [33]. Recombinant BB0363 was shown to have c-di-GMP phosphodiesterase activity. The *bb0363* mutant, which likely has high intracellular levels of c-di-GMP, was found to be defective in motility *in vitro* [33]. *In vivo*, the *bb0363* mutant was able to survive in ticks but failed to establish infection in mice, suggesting that high levels of c-di-GMP are detrimental for spirochetes to replicate in a mammalian host. However, whether c-di-GMP is required for any stage of the infectious cycle of *B. burgdorferi* remains undetermined. In this study, we generated an *rrp1* mutant in the infectious clone of *B. burgdorferi*, B31 5A4NP1. We show that Rrp1 is dispensable for mammalian infection but is essential for spirochetal survival in the tick vector. We further show that the Rrp1 requirement is, in part, due to its control over the expression of glycerol transport and metabolism in *B. burgdorferi*.

## Results

### Generation of the *rrp1* mutant and the repaired strain

**Figure 1:**
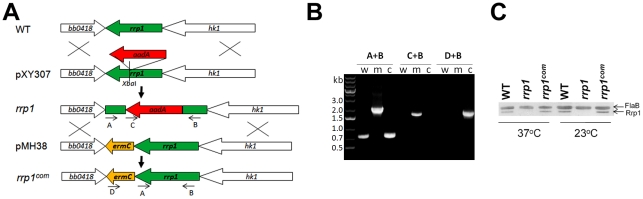
Construction of the *rrp1* mutant and the repaired strain. (**A**) Strategy for construction of the *rrp1* mutant and the repaired strain (*rrp1^com^*). Arrows indicate the approximate positions of the oligonucleotide primers used for PCR analysis. (**B**) PCR analysis of strains. The specific primer pairs used in PCR are indicated above lanes. Lane W, wild-type (5A4NP1); lane M, the *rrp1* mutant; lane C, *rrp1^com^*. (**C**) Western blot analysis of whole-cell lysates of WT, *rrp1*, and *rrp1^com^* spirochetes probed with α-Rrp1 and α-FlaB monoclonal antibodies.

To determine the role of c-di-GMP in *B. burgdorferi* pathogenesis, we constructed an *rrp1* mutant in the infectious *B. burgdorferi* strain 5A4NP1 (See **Table 1** for a list of strains used in this study). This was accomplished by replacing the wild-type chromosomal *rrp1* copy with a disrupted gene via homologous recombination (**Fig. 1A**). A similar approach was used to repair the wild-type *rrp1* gene by replacing the mutated copy with a wild-type *rrp1* (**Fig. 1A**). The genotypes of the *rrp1* mutant and the repaired strain (*rrp1^com^*) were confirmed by PCR (**Fig. 1B**) and by immunoblot analyses (**Fig 1C**).

**Table 1:** *B. burgdorferi* strains used in this study.

Strains	Description	Sources
5A4NP1	wild-type B31 with *bbe02* disrupted with a *kan* marker	[87]
*rrp1*	5A4NP1 with *rrp1* disrupted with an *aadA* marker	This study
*rrp1^com^*	*the rrp1 mutant* repaired with a wild-type copy of *rrp1* linked to an *ermC* marker	This study
*glp*	5A4NP1 with *bb0240-bb0243* disrupted with an *aacC* marker	This study
*glp^com^*	the *glp* mutant repaired with a wild-type copy of *bb0240-bb0243* linked to an *aadA* marker	This study
*rrp1/flaBp-glp*	the *rrp1* mutant carrying a copy of *bb0240-bb0243* driven by a *flaB* promoter	This study

### Rrp1 is not required for spirochetal infection in mammals

To determine the role of c-di-GMP in mammalian infection, we needle-inoculated groups of mice with various *B. burgdorferi* strains (10^5^ spirochetes/mouse). Two-weeks post inoculation, ear punch biopsies were cultured in BSKII medium for the presence of spirochetes. Similar to wild-type spirochetes, the *rrp1* mutant was readily detected in either immunocompetent (C3H/HeN) or immunocompromised (C3H-SCID) mouse strains (**Table 2**). No major difference in ID_50_ values between wild-type and the *rrp1* mutant (**Table 3**). Further analysis of histopathology revealed that the *rrp1* mutant elicited Lyme arthritis similar to that induced by wild-type *B. burgdorferi*. (**Supplemental Fig. S1**). This result indicates that abrogation of c-di-GMP synthesis does not affect the ability of *B. burgdorferi* to infect mice. We conclude that c-di-GMP is dispensable for mammalian infection. This is in contrast with an avirulent phenotype observed in *B. burgdorferi* lacking the c-di-GMP phosphodiesterase BB0363 [33].

**Table 2:** Mouse infectivity of the *rrp1* mutant.

	No. of mice infected/total No. of mice			
	Needle infection (10^5^ spirochetes/mouse)		Tick infection	
	C3H/HeN	C3H-SCID	Natural	Microinjection
WT	10/10	9/9	9/9	9/9
*rrp1*	12/12	9/9	0/9	0/9
*rrp1^com^*	NA	9/9	9/9	9/9

**Table 3:** ID_50_ values of various *B. burgdorferi* strains.

	No. of mice infected/total No. of C_3_H/HeN mice				
	3×10	3×10^2^	3×10^3^	3×10^4^	ID_50_
WT	0/5	0/5	3/5	5/5	2.0×10^3^
*rrp1*	0/5	0/5	2/5	5/5	4.3×10^3^
*glp*	0/5	0/5	1/5	5/5	7.1×10^4^
*glp^com^*	0/5	1/5	5/5	5/5	7.1×10^2^

### Rrp1 is essential for spirochetal survival in ticks

**Figure 2:**
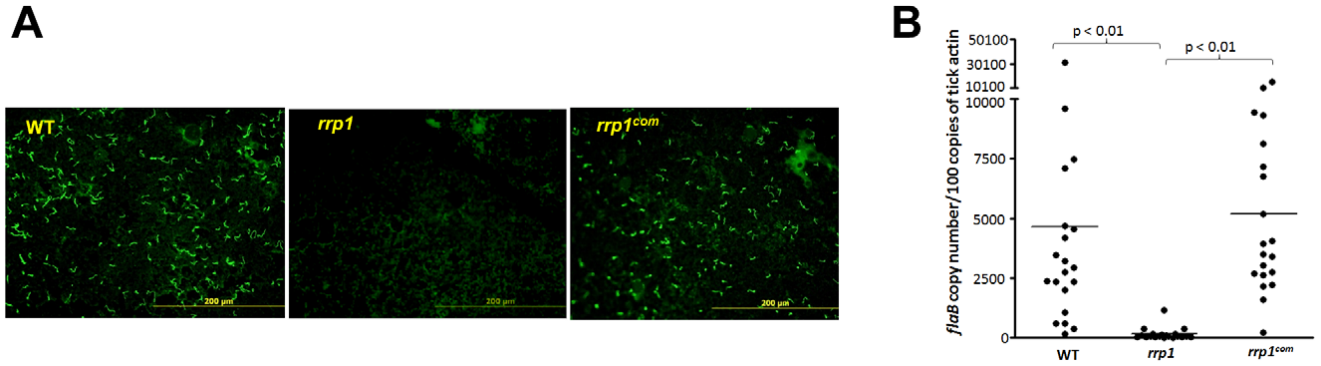
The *rrp1* mutant failed to survive in ticks upon acquisition. (**A**) IFA analysis of spirochetes from fed larvae. *I. scapularis* larvae were fed on needle infected C3H/SCID mice harboring wild-type (5A4NP1), *rrp1*, or *rrp1^com^* spirochetes. Engorged larvae were collected after repletion and subjected to IFA analysis using fluorescein isothiocyanate-labeled anti-*B. burgdorferi* antibody. Twelve ticks were examined in each group and a representative image for each group of ticks is shown. (**B**) qPCR analyses of spirochete burden in fed larvae. Quantitative PCR for the *B. burgdorferi flaB* gene was performed with DNA extracted from fed larvae. Each dot represents one data point from one three larval tick (a total of 20 samples with 60 ticks examined for each group from two independent experiments). The horizontal bar represents the mean value of each group.

To examine the *rrp1* mutant's phenotype in the tick cycle, groups of pathogen-free *Ixodes scapularis* larvae were fed on C3H/SCID mice that were needle-infected with the wild-type, *rrp1^mut^* or *rrp1^com^* strains two weeks after infection. Engorged larvae were collected after repletion and tick contents were subjected to immunofluorescence assay (IFA). In contrast to the wild-type and *rrp1^com^* strains that were readily detectable in fed larvae, virtually no *rrp1* mutant spirochetes were observed (**Fig. 2A**). Further quantitative PCR analysis revealed that there were significantly lower numbers of the *rrp1* mutant than that of wild-type or *rrp1^com^* strains in ticks (**Fig. 2B**).

**Figure 3:**
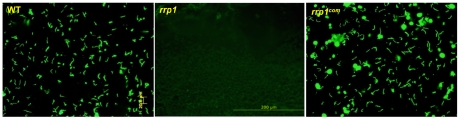
The *rrp1* mutant could not survive in artificially infected ticks upon feeding. Unfed *I. scapularis* nymphs were microinjected with wild-type (5A4NP1), *rrp1*, or *rrp1^com^* spirochetes and then fed on naïve mice. The engorged nymphs were collected after repletion and subjected to IFA analysis using fluorescein isothiocyanate-labeled anti-*B. burgdorferi* antibody. Ten ticks were examined in each group and a representative image for each group of ticks is shown in this figure.

The inability to detect the *rrp1* mutant in tick midguts after feeding could be due either to a defect in tick midgut survival or a defect in migration from the mouse to the tick. To test these two possibilities, we used microinjection to directly place spirochetes into midguts of nymphal ticks [34], [35]. These artificially infected ticks then fed on naïve mice. Detached ticks were collected and subjected to IFA analysis. As shown in **Fig. 3**, the wild-type and *rrp1^com^* strains were readily detectable in ticks, whereas the *rrp1* mutant remained undetected.

To confirm that the *rrp1* mutant is defective in the ability to survive in ticks, engorged larvae that were fed on infected mice from the experiments described above were allowed to molt to nymphs in an environmental chamber. Unfed nymphs were then fed on naïve mice. Ticks that were infected with either the wild-type or *rrp1^com^* strains could readily infect naïve mice, whereas ticks infected with the *rrp1* mutant could not (**Table 2**). Similarly, ticks that were artificially infected with the *rrp1* mutant were also unable to infect C3H/SCID mice (**Table 2**). These results support the notion that the *rrp1* mutant is unable to survive in the tick vector.

### Transcriptome analyses of the *rrp1* mutant

To investigate the molecular mechanisms underlying the requirement of c-di-GMP for spirochete survival in ticks, we sought to identify genes whose expression was affected by the deletion of *rrp1*. To do so, we performed two independent microarray analyses: one comparing transcriptional profiles of the wild-type and *rrp1* mutant and the other comparing transcriptional profiles of the *rrp1* mutant and the *rrp1^com^* strain. The comparison of the transcriptomes of the wild-type and *rrp1* mutant revealed 120 genes whose expressions were up- or down-regulated by Rrp1 (cut-off >3-fold) (**Text S1**). Among these, 39 genes whose dependence on Rrp1 could be confirmed by the comparison of the transcriptomes of the *rrp1* and *rrp1^com^* strains (cut-off >3-fold) (**Table 4**). We considered these genes to be the most reliable candidates for Rrp1-dependent regulation.

**Table 4:** Rrp1-regulated genes in *B. burgdorferi.*

Locus	Common Name	WT/*rrp1* *	*rrp1^com^*/*rrp1* **
BBP27	rev protein	21.46	4.42
BBM27	rev protein	19.36	4.17
BBN28	MlpI, lipoprotein	18.62	12.35
BBM28	MlpF, lipoprotein	15.09	12.82
BBM38	erpK protein	12.12	23.81
**BB0241**	**glycerol kinase**	**7.74**	**9.35**
BBF23	conserved hypothetical protein	7.57	0.04
**BB0242**	**hypothetical protein**	**6.92**	**11.36**
BBA07	chpAI protein, putative (homolog to Mlp)	6.70	7.46
**BB0243**	**glycerol-3-phosphate dehydrogenase**	**6.48**	**8.26**
**BB0240**	**glycerol uptake facilitator**	**5.53**	**5.49**
BBA33	hypothetical protein	5.23	13.33
BBO31	conserved hypothetical protein	4.42	3.24
BBJ23	hypothetical protein	4.35	12.20
BBL39	erpA protein	4.14	3.53
BBL30	conserved hypothetical protein	4.14	6.29
BBL31	conserved hypothetical protein	4.12	3.14
BB0844	hypothetical protein	3.79	4.17
BBO30	conserved hypothetical protein	3.76	3.95
BBP28	MlpA, lipoprotein, MlpA	3.50	3.92
BBO37	conserved hypothetical protein	3.35	5.59
BB0322	hypothetical protein	3.33	11.24
BBB11	conserved hypothetical protein	3.31	5.92
BBA73	antigen, P35, putative	3.30	16.13
BBJ01	hypothetical protein	3.25	7.63
BBL36	conserved hypothetical protein	3.23	5.99
BBR40	erpH protein	3.23	10.53
BBR36	conserved hypothetical protein	3.20	6.76
BBN42	hypothetical protein	3.16	3.60
BBM35	conserved hypothetical protein	3.12	9.26
BBB19	ospC, outer surface protein C	3.03	3.09
BBP05	hypothetical protein	0.33	0.31
BBK41	hypothetical protein	0.33	0.30
BBA56	hypothetical protein	0.31	0.13
BB0467	conserved hypothetical protein	0.27	0.32
BBA22	hypothetical protein	0.25	0.20
BBU03	hypothetical protein	0.20	0.14
BBJ15	hypothetical protein	0.17	0.30
BBU12	conserved hypothetical protein, authentic frameshift	0.10	0.33

*fold changes in gene expression between wild-type and the *rrp1* mutant.

**fold changes in gene expression between the complemented strain and the *rrp1* mutant.

### Rrp1 controls expression of glycerol transport and metabolism

Genes regulated by Rrp1 are distributed throughout the genome and extra-chromosomal segments of *B. burgdorferi* (**Table 4**, Locus numbers start with BB and a letter are extra-chromosomal genes [24]). Among these genes, an intriguing target of Rrp1 regulation was an apparent *glp* operon encoding glycerol transport/metabolism genes, *bb0240*-*bb0243* [24], [25], [36], [37]. The first gene of the operon, *bb0240*, encodes a putative glycerol uptake facilitator (GlpF), followed by a putative glycerol kinase gene (*bb0241, glpK*), a small putative hypothetical gene (*bb0242*), and a putative glycerol-3-phosphate dehydrogenase gene (*bb0243, glpA/glpD*). Glycerol can be utilized in energy production as a biosynthetic precursor to membrane lipids or lipoproteins [24], [25], [36], [37].

**Figure 4:**
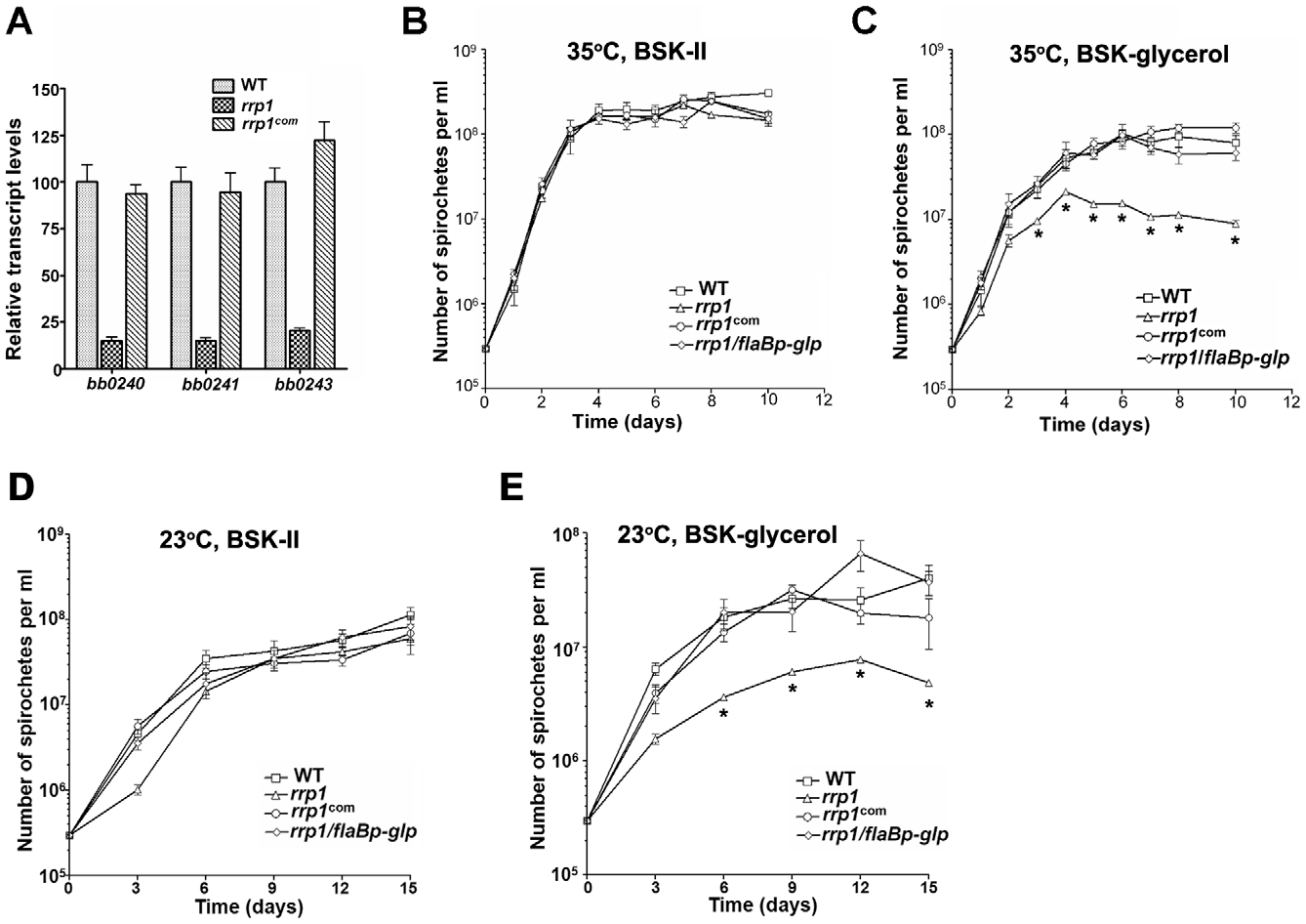
Rrp1 controls expression of the glycerol gene operon (*bb0240-0243)*. (**A**) Relative transcript levels of the glycerol operon *glp* (*bb0240-bb0243*) in wild-type, *rrp1*, and *rrp1^com^* by real-time RT-PCR. RNA was isolated from late logarithmic phase cultures grown at 35°C in standard BSK-II medium. Values represent the average copy number for each gene (± standard deviation) normalized per 1000 copies of *flaB.* (**B–E**) growth curves of wild-type, *rrp1*, *rrp1^com^* or *rrp1/flaBp-glp* at 23°C (**D, E**) or 35°C (**B, C**) in standard BSK-II medium (**B, D**) or BSK-glycerol medium (**C, E**). The initial cell density was 3×10^5^ cells/ml for each strain. Spirochetes were enumerated under dark-field microscopy. Data presented here is from one representative experiment with three independent cultures. Each data point was the average of data from three independent cultures. *, P<0.05.

qRT-PCR analysis confirmed that induction of *bb0240-bb0243* was indeed under the control of Rrp1 (**Fig. 4A**). We hypothesized that if *bb0240-bb0243* is involved in glycerol transport and metabolism, the *rrp1* mutant may have a defect in the utilization of glycerol as a carbon source. To test this hypothesis, the wild-type, *rrp1* and *rrp1* ^com^ strains were cultivated in either standard BSKII medium or in a modified BSKII medium where glucose was replaced with glycerol (BSK-glycerol, which was prepared from glucose free CMRL) [37]. The *rrp1* mutant was not impaired in growth in the standard BSKII medium at either 35°C (**Fig. 4B**) or 23°C (**Fig. 4D**). However, when grown in the BSK-glycerol medium, the *rrp1* mutant failed to reach the cell density of the wild-type or *rrp1* ^com^ (**Fig. 4C and 4E**). Thus, glycerol transport and metabolism appeared to be particularly important at later time points in the growth curve. BSK medium is a complex medium containing many undefined components including rabbit serum and BSA as well as other potential carbon source such as pyruvate. Presence or absence of pyruvate did not significantly affect the growth of either wild-type or the *rrp1* mutant in BSK-II or BSK-glycerol medium (data not shown). BSK-glycerol medium also contains 0.1 g/L of glucose, determined by D-Glucose Kit (Roche Applied Science, Indianapolis, IN), which may contribute to the initial growth of the *rrp1* mutant in BSK-glycerol medium (the standard BSK-II medium contains 6 g/L of glucose). Nevertheless, these experiments verified the involvement of Rrp1 in glycerol transport/metabolism.

### Glycerol enhances expression of *rrp1*

**Figure 5:**
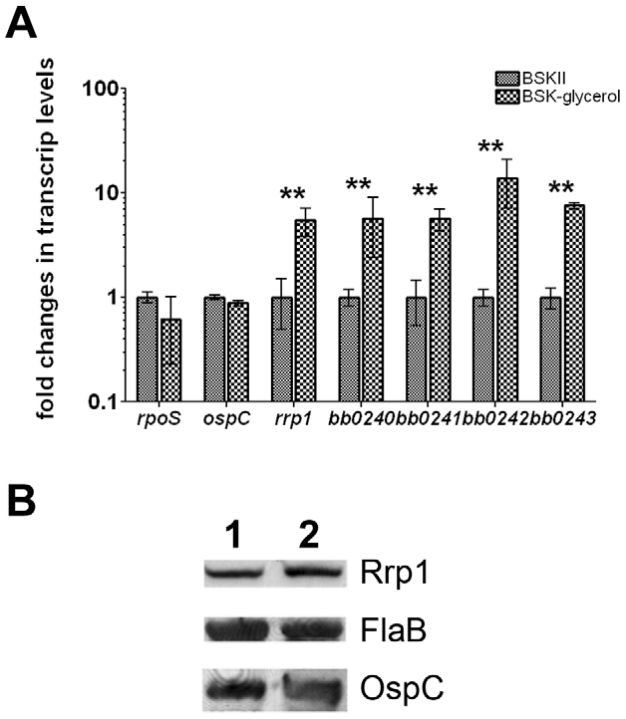
Glycerol induces expression of *rrp1* and *bb0240-bb0243*. Wild-type *B. burgdorferi* strain B31 5A4NP1 was grown at 35°C in either standard BSKII or BSK-glycerol medium. Cells were harvested at late logarithmic phase. (**A**) qRT-PCR. RNAs were extracted and subjected to real-time RT-PCR analyses for *rrp1*, *bb0240*, *bb0241*, *bb0242*, *bb0243*, *rpoS*, *ospC*, and *flaB*. Levels of expression of each gene were normalized with the level of *flaB* expression in each sample. Relative fold change of gene expression between the two growth conditions were reported (with levels of expression of each gene in standard BSKII media as values of 1). **, p<0.01. (**B**) immunoblot against Rrp1, FlaB, or OspC. Lane 1, spirochetes cultivated in BSK-II medium. Lane 2, spirochetes cultivated in BSK-glycerol medium. Upon normalization against FlaB, Rrp1 level is 1.7 fold higher in BSK-glycerol medium than that in standard medium.

We further tested the possibility that expression of *rrp1* is also influenced by glycerol. RNA was extracted from wild-type *B. burgdorferi* grown in either standard BSKII or BSKII-glycerol medium. The extracted RNAs were subjected to qRT-PCR analysis. Growth in the BSKII-glycerol medium did not significantly alter expression of Rrp2-dependent genes such as *rpoS* and *ospC*. However, the transcript level of *rrp1* as well as the glycerol metabolic genes *bb0240-bb0243* were dramatically upregulated when grown in BSKII-glycerol medium (**Fig. 5A**). However, Rrp1 protein level is much less influenced by this growth condition (1.7 fold) (**Fig. 5B**). Nevertheless, this observation suggests that glycerol may potentially enhance *rrp1* expression.

### Glycerol transport and/or metabolism is required for maximal spirochete burden in ticks

**Figure 6:**
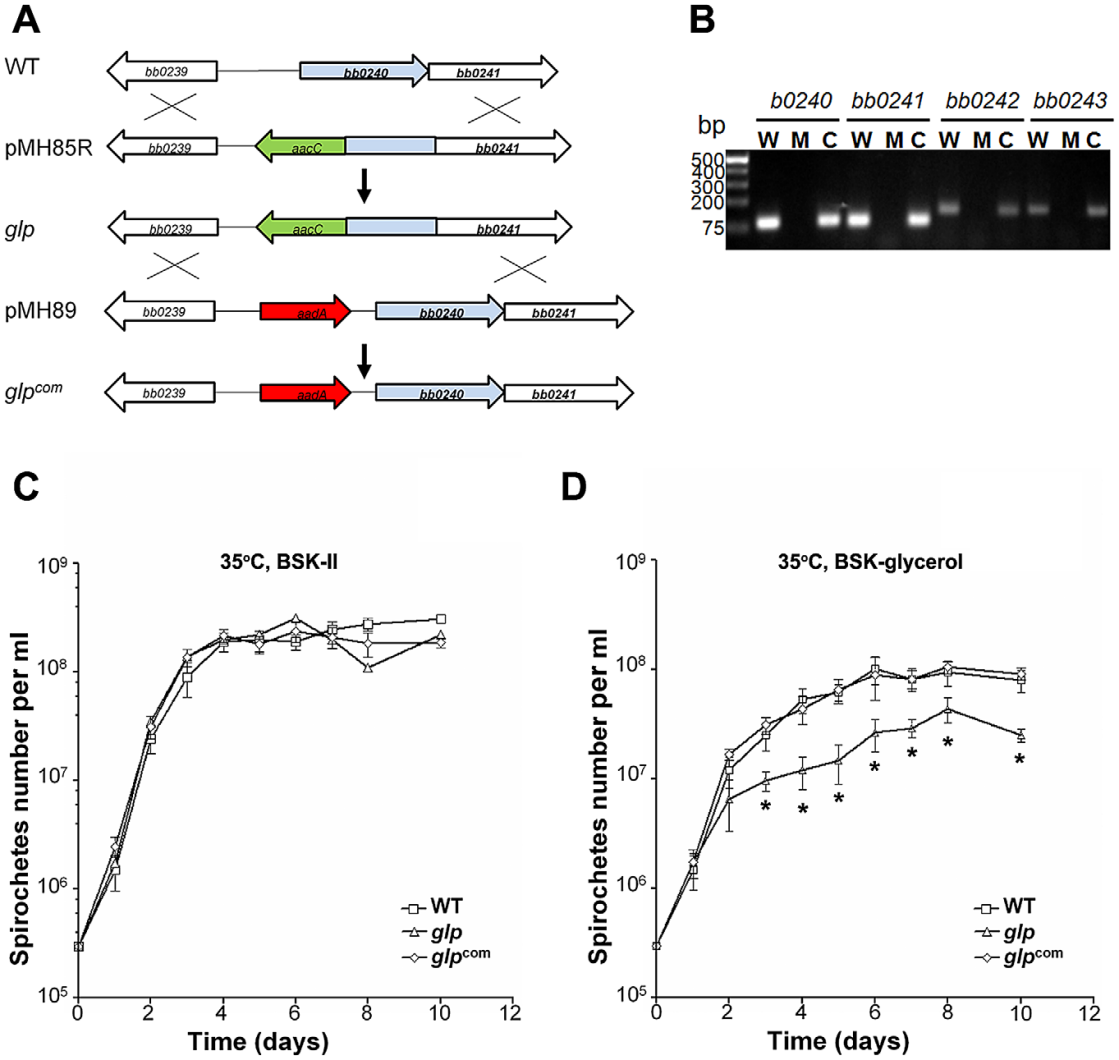
Construction of the *glp* mutant and the repaired strain. (**A**) Strategy for construction of the *glp* mutant and the repaired strain (*glp^com^*). (**B**) RT-PCR analysis of expression of *bb0240-bb0243*. RNA was isolated from late logarithmic phase cultures grown at 35°C in the BSK-II medium (pH 7.5). W: wild-type strain 5A4NP1; M: the *glp* mutant; C: the repaired strain (*glp^com^*). (**C**) and (**D**) Growth curve of wild-type, *glp* and *glp^com^* at 35°C in standard BSK-H medium (C) or BSK-glycerol medium (D). The initial cell density was 3×10^5^ cells/ml for each strain. Spirochetes were enumerated under dark-field microscopy. Data presented here is from one representative experiment with three independent cultures. Each data point was the average of data from three independent cultures. *, p< 0.05.

Because Rrp1 was required for full induction of the glycerol operon and for maximal growth in the BSKII-glycerol medium, we hypothesized that defective glycerol metabolism by the *rrp1* mutant could contribute to the mutant's inability to survive in ticks. If so, a mutant defective in glycerol metabolism would be expected to have a phenotype similar to that of the *rrp1* mutant. To test this hypothesis, we constructed a *glp* mutant by deleting a portion of the first gene *bb0240* and its upstream promoter of the *bb0240-bb0243* operon (**Fig. 6A**). qRT-PCR analysis confirmed that the *glp* mutant lacks *bb0240 bb0241*, *bb0242*, and *bb0243* mRNA (**Fig. 6B**). Expression of *bb0240-bb0243* was restored when the mutated *bb0240* gene and the promoter region was replaced by the wild-type copy of *bb0240* at the native location (designated as *glp^com^*. **Fig. 6A and 6B**).

We first examined the growth phenotype of the *glp* mutant *in vitro*. The mutant had no detectable growth defect when grown in standard BSKII medium (**Fig. 6C**). However, similar to the *rrp1* mutant, the *glp* mutant could not reach the same cell density as the parent wild-type strain when grown in the BSK-glycerol medium (**Fig. 6D**). This defect resulted from abrogation of *bb0240-0243* expression, as the growth defect was readily restored upon restoration of *bb0240-bb0243* expression in *glp^com^* (**Fig. 6A & 6D**). This result is consistent with the prediction that the growth defect of the *rrp1* mutant in the BSK-glycerol medium is due to the loss of expression of *bb0240-bb0243*.

**Figure 7:**
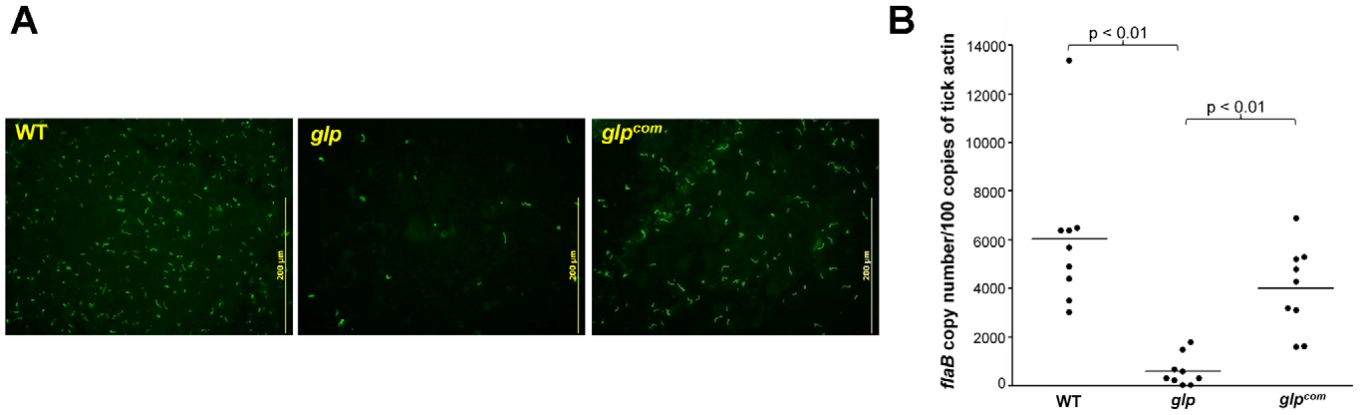
Glycerol transport/metabolism is important during tick residence. Naïve larvae fed on needle inoculated C3H/HeN mice infected with wild-type, *glp*, or *glp^com^*. Fed larvae were allowed to molt to nymphs. Infected unfed nymphs were then fed on naïve mice to produce fed nymphs. Fed nymphs were then subjected to IFA analysis (**A**) and qPCR analysis (**B**). For qPCR, copies of the *B. burgdorferi flaB* genes were chosen to represent spirochete numbers and the values were reported relative to 100 copies of the tick actin gene. Each data point is from one tick.

We then examined the phenotype of the *glp* mutant *in vivo*. The wild-type, *glp* mutant or glp*^com^* spirochetes (10^5^ spirochetes/mouse), were intradermally inoculated into C3H/HeN mice. Two weeks after inoculation, ear punch biopsies from all mice were culture-positive for spirochetes, suggesting that BB0240-BB0243 are not required for mammalian infection (**Table 5**). Further determination of the ID_50_ values showed that the *glp* mutant has a slight infectivity deficit relative to wild-type *B. burgdorferi*, with 1-log-unit increase in the ID_50_ (**Table 3**). To examine the role of *bb0240-0243* in the tick-mouse cycle, pathogen-free unfed larvae were placed on infected mice. Fed larvae were collected and allowed to molt to nymphs. Unfed nymphs then fed on groups of naïve C3H/HeN mice. Ticks at various stages were collected for IFA and/or qRT-PCR analyses. We observed that although detectable in ticks, the *glp* mutant had reduced spirochetal loads compared to the wild-type or *glp^com^* strains (**Fig. 7A & 7B**, only results from nymphs were shown). These data suggest that, similar to Rrp1, the glycerol transport/metabolic pathway is required for the optimal colonization of *B. burgdorferi* in ticks and that the loss of *bb0240-bb0243* expression in the *rrp1* mutant contributes to its poor survival in ticks.

**Table 5:** Mouse infectivity of the *glp* mutant.

	No. of mice infected/total No. of mice	
	Needle infection (10^5^ spirochetes/mouse)	Tick infection
WT	5/5	6/6
*glp*	5/5	3/6
*glp^com^*	5/5	4/6

Mice two weeks post tick feeding were also examined for the presence of spirochetes in various tissue samples (skin, heart, and joint). Unlike the *rrp1* mutant that failed to infect mice via tick bites, the *glp* mutant was capable of completing the tick-mouse cycle and subsequently infecting naïve mice upon tick feeding (**Table 5**), despite its reduced survival in ticks. Note that both the *glp* mutant and *glp^com^* strains showed partially reduced infectivity via tick bites, indicating that this reduction of infectivity is not due to the loss of *bb0240-bb0243* (**Table 5**). These results indicate that loss of *bb0240-bb0243* expression of the *rrp1* mutant could not fully account for the inability of the *rrp1* mutant to complete its enzootic cycle and that Rrp1 controls additional factor(s) involved in the spirochetal life cycle in ticks.

### Constitutive expression of glycerol metabolic pathway partially rescues the *rrp1* mutant's phenotype in ticks

**Figure 8:**
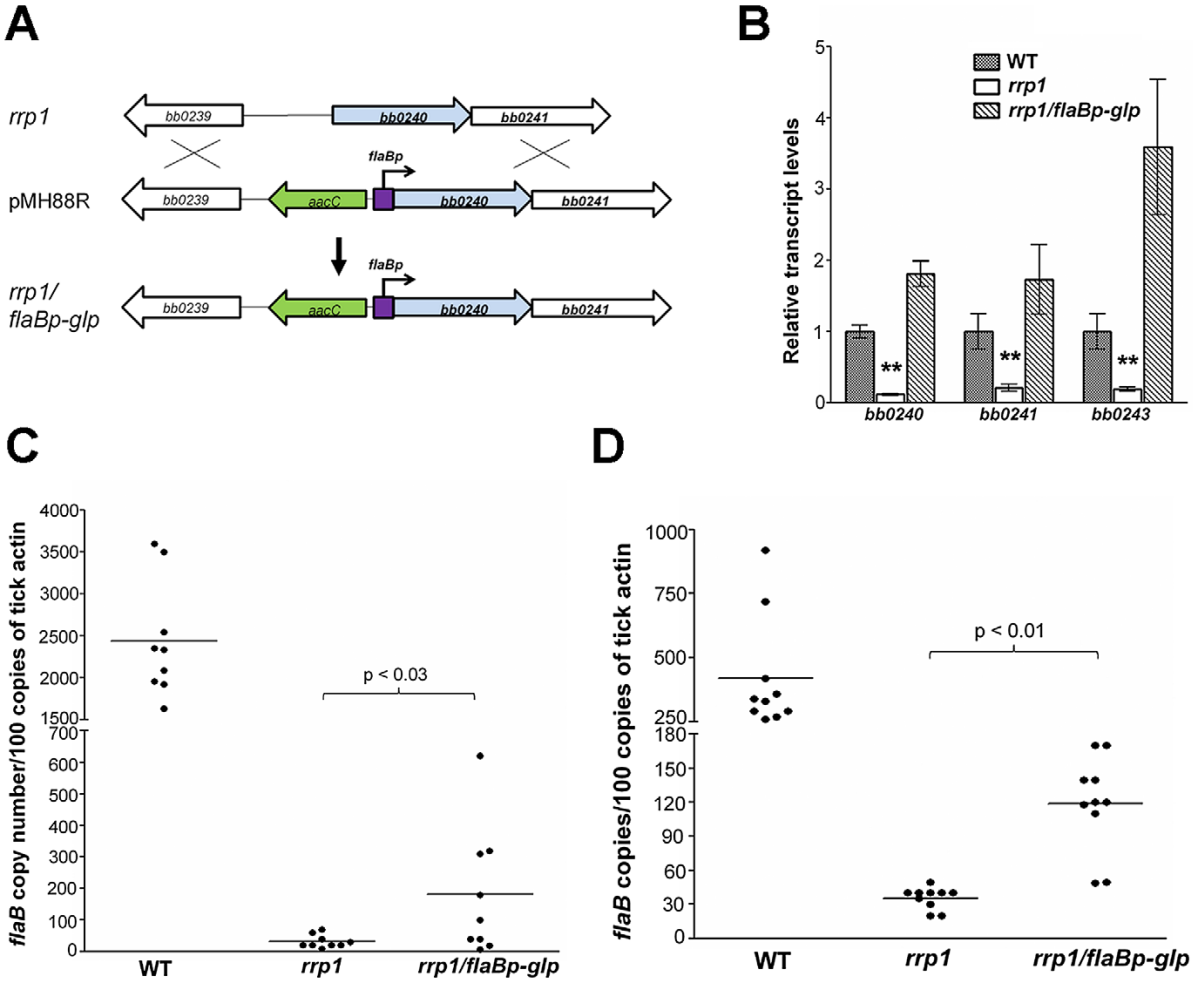
Constitutive expression of glycerol metabolism genes in the *rrp1* mutant partially restored spirochetal survival in ticks. (**A**) Strategy for construction of an *rrp1* mutant with constitutive expression of *bb0240-bb0243*, by replacing the promoter region of *bb0240* with the *flaB* promoter. (**B**) Real-time RT-PCR analysis of expression of *b0240, bb0241*, and *bb0243*. RNA was isolated from late logarithmic phase cultures of wild-type (WT), the *rrp1* mutant (*rrp1*), or *rrp1/flaBp-glp* that had been grown at 35°C in the BSK-II medium (pH 7.5). Levels of gene expression were normalized with the level of *flaB* gene expression. Fold changes of gene expression relative to wild-type strain (set as 1) were reported. (**C**) and (**D**), qPCR analysis of spirochetal burden in fed larvae one day (**C**) or fourteen days (**D**) after repletion. Copies of the *B. burgdorferi flaB* genes were chosen to represent spirochete numbers and are normalized with 100 copies of the tick actin gene in each sample. Each data point is from three fed larval ticks. **, p<0.01.

To further investigate the role of glycerol transport and metabolism during tick infection, we constitutively expressed the *bb0240-bb0243* operon in the *rrp1* mutant using an independent *flaB* promoter (**Fig. 8A**). The resulting strain, designated as *rrp1^mut^/flaBp-glp*, expressed *bb0240-bb0243* in an Rrp1-independent fashion (**Fig. 8B**) and fully rescued the growth defect of the *rrp1* mutant in the BSK-glycerol medium (**Fig. 4B & 4C**). This observation provides additional genetic evidence that the growth defect of the *rrp1* mutant is due to impaired glycerol transport/metabolism.

To compare the phenotypes in ticks, the wild-type, *rrp1* mutant, or *rrp1/flaBp-glp* strains were needle-infected into naïve mice. Unfed larvae were allowed to feed on these infected mice. qPCR analyses on fed larvae showed that the *rrp1^mut^/flaBp-glp* spirochetes had a 4- to 5-fold increase in spirochetal load compared to the load of the *rrp1* mutant (**Fig. 8C & 8D**). This increase suggests that restoration of expression of glycerol transport/metabolism can improve survival of the *rrp1* mutant in ticks. However, the spirochete load of *rrp1/flaBp-glp* was still drastically lower than that of wild-type spirochetes (**Fig. 8C & 8D**). To determine if the *rrp1/flaBp-glp* spirochetes are able to migrate to mice, fed larvae were collected and allowed to molt to nymphs. Infected nymphs were then used to infect naïve C3H/SCID mice. The result showed that, similar to the *rrp1* mutant, the *rrp1^mut^/flaBp-glp* strain was incapable of completing the tick-mouse cycle to infect naïve mice (**Table 6**). These data further support the conclusions that while glycerol transport/metabolism is important during tick residence, additional Rrp1-dependent factor(s) are involved in the tick-mouse cycle of *B. burgdorferi*.

**Table 6:** Mouse infectivity of the *rrp1* mutant with constitutive expression of *bb0240-bb0243.*

	No. of mice infected/total No. of mice		
	Needle infection	Tick infection	
		Natural	Microinjection
wild type	4/4	4/4	5/5
*rrp1*	4/4	0/4	0/5
*rrp1/flaBp-glp*	4/4	0/4	0/3

## Discussion

During the transmission process between mammals and ticks, *B. burgdorferi* dramatically alters the expression of many genes that are essential for spirochete survival in either host (for reviews, see [31], [38]). In the past few years, we and others have shown that one of the *B. burgdorferi* two-component signaling systems, Hk2-Rrp2, functions as a key signaling pathway that governs expression of genes necessary for mammalian host infection [26], [27], [29], [30], [39]. In this study, we provide genetic evidence that the other two-component system, Hk1-Rrp1, is dispensable for mammalian infection, yet plays a vital role in the tick, in part, by controlling expression of the glycerol transport/metabolic pathway of *B. burgdorferi*.

Rrp1 is a diguanylate cyclase responsible for synthesis of the second messenger c-di-GMP [8], [32]. The importance of c-di-GMP to bacterial pathogenesis has been well documented [4], [21], [22]. In many cases, the impact of c-di-GMP on pathogenesis is due to its effect on biofilm formation or motility [40], [41], [42], [43]. An interesting example that is related to this study involves another vector-borne pathogen, *Yersinia pestis*. Similar to the phenotype of the *rrp1* mutant in ticks that we have described herein, disruption of *hmsT*, a gene encoding diguanylate cyclase in *Y. pestis*, reduces the transmission of plague bacteria from fleas to mammals [44], [45], [46], [47]. However, the mechanisms of influencing transmission by c-di-GMP in these two pathogens seem to be different. Inactivation of *hmsT* results in a defect in biofilm formation but not in replication of *Y. pestis* in fleas, which is important for the spread of *Y. pestis* from fleas to mammals. Currently there is no evidence that *B. burgdorferi* forms biofilms. The *B. burgdorferi* genome encodes a *luxS* gene responsible for the autoinducer AI2 synthesis, which is necessary for biofilm formation in some bacteria [48], [49], [50]. However, inactivation of *luxS* does not affect the life cycle of *B. burgdorferi* in either ticks or mice [51], [52]. Therefore, the mechanism of action of c-di-GMP in the enzootic cycle of *B. burgdorferi* is different from that of *Y. pestis*.

In addition to affecting biofilm formation and motility, c-di-GMP modulates many other activities that may not be related to multicellular behavior such as cell division, phage resistance, heavy metal resistance, etc. [2], [3], [4]. With regards to bacterial pathogenesis, c-di-GMP has been reported to affect the processes of adhesion, invasion, and toxin production by modulating the production or activities of virulence factors [21], [22], [53], [54], [55]. However, modulation of bacterial infection by the control of glycerol metabolism is observed here for the first time. In this study, we provide the following lines of evidence supporting the notion that c-di-GMP controls glycerol transport and metabolism in *B. burgdorferi*, which in turn is important for its survival in ticks. 1) Expression of *bb0240-bb0243* is significantly downregulated by abrogation of Rrp1 (**Table 4** & **Fig. 4A**). 2) Both the *rrp1* mutant and the *glp* mutant show growth defects in BSK-glycerol medium (**Fig. 4C** & **Fig. 6D**). 3) The *glp* mutant has reduced survival in ticks (**Fig. 7**). 4) Restoration of *bb0240-bb0243* expression in the *rrp1* mutant rescues the growth defect *in vitro* and enhances the survival of the *rrp1* mutant in ticks.

What roles does glycerol transport/metabolism play in *B. burgdorferi* physiology? As an obligate pathogen, *B. burgdorferi* has a reduced genome and lacks many metabolic pathways such as the TCA cycle and those for synthesis of amino acids, nucleotides, and fatty acids [24], [25], [36]. *B. burgdorferi* does encode proteins for the utilization of several sugars in addition to glucose [24], [25], [36], [37]. Notably, a complete pathway for transport and utilization of glycerol (BB0240-BB0243) is preserved. Bioinformatics analysis suggests that upon uptake of glycerol (by glycerol uptake facilitator BB0240, GlpF), glycerol is converted to glycerol-3-P by glycerol kinase (BB0241, GlpK) [36], [37]. Glycerol-3-P can either feed into lipid/lipoprotein biosynthesis or enter the ATP-generating stage of glycolysis via conversion to glyceraldehyde 3-phosphate by glycerol-3-P dehydrogenase (BB0243, GlpA/GlpD) and triosephosphate isomerase (BB0561) [36], [37]. In other words, the glycerol and glucose pathways interconnect, and glycerol can be an important carbon and energy source at times when glucose becomes limited. This notion is supported by a previous study [37] as well as the *in vitro* growth data from this study (**Fig. 4B & 4C**). Based on the observation that the glycerol pathway-defective *glp* mutant replicates normally in mice but has reduced growth in ticks, we postulate that *B. burgdorferi* utilizes different carbon/energy sources within each host environment. During mammalian infection when glucose is readily available (0.1–0.2% in mouse blood) [56], *B. burgdorferi* utilizes glucose as the main carbon and energy source. Thus, inactivation of the *glp* operon does not dramatically affect spirochete replication in mammals. When spirochetes enter the tick vector, initially the *glp* mutant may be able to replicate with the presence of glucose from blood. Then, glucose may become limiting, while glycerol, on the other hand, may be available in ticks. This notion is consistent with the fact that the *glp* mutant remains capable of surviving in ticks but with reduced spirochetal numbers (**Fig. 7**). It is noteworthy that many insects including ticks produce glycerol as an anti-freezing molecule [57]. Therefore, activation of the glycerol transport and metabolism via Rrp1 could ensure optimal growth of *B. burgdorferi* in the tick vector. Further, growth on glycerol appears to provide a positive feedback on *rrp1* gene expression (**Fig. 5**).

How does c-di-GMP control the expression of *bb0240-bb0243*? One of the characterized mechanisms employed by c-di-GMP to influence gene regulation is through a unique riboswitch RNA structure. It was shown that c-di-GMP can directly bind to a riboswitch located in the 5′ UTR region of target genes and can influence gene transcription and/or translation [58]. We did not find c-di-GMP-specific riboswitches upstream of *bb0240*. C-di-GMP can also modulate gene expression by affecting expression or activity of transcription factors [59], [60], [61], [62], [63]. Some of these transcription factors bind c-di-GMP directly [64], [65]. Interestingly, c-di-GMP controls DNA binding of a subgroup of CRP (cAMP receptor protein) transcription factors that activate genes involved in utilization of alternative carbon and energy sources (other than glucose). For example, Clp, a CRP homolog from *Xanthomonas campestris* binds c-di-GMP and regulates virulence gene expression [65], [66], [67]. In *Vibrio cholerae*, it was shown that cAMP-CRP controls expression of a DGC that, in turn, governs the production of c-di-GMP and biofilm formation [68]. Bioinformatic analysis did not identify any CRP homologue encoded in the *B. burgdorferi* genome. Recently, it was reported that another transcriptional regulator in *B. burgdorferi*, BosR, also affects *glp* expression [69], [70]. Thus, it is possible that c-di-GMP may influence *glp* via BosR. Nevertheless, elucidating the mechanism of how Rrp1 controls expression of the glycerol pathway in *B. burgdorferi* will shed light on the interplay between c-di-GMP and carbon utilization networks.

Work on Rrp1 from this study and previous studies [8], [32] strongly supports the notion that c-di-GMP is essential for spirochetal adaptation in the tick vector but is not required for mammalian infection. In fact, c-di-GMP is not only dispensable, shutting down the synthesis of c-di-GMP is necessary for *B. burgdorferi* to successfully establish infection in the mammalian host. This was recently demonstrated by Sultan *et al*., when they showed that the *B. burgdorferi* mutant missing c-di-GMP phosphodiesterase (BB0363) failed to infect mice [33]. This is consistent with an emerging theme that uncontrolled production of c-di-GMP is detrimental to the acute phase of bacterial infection [3], [21], [22]. Thus, a tight regulation of the synthesis of c-di-GMP is important for *Borrelia* adaptation in both the tick vector and the mammalian host.

What are the downstream effectors of c-di-GMP in *B. burgdorferi*? The *bb0363* mutant showed a defect in motility, suggesting that flagellar proteins or gene transcription of *B. burgdorferi* may be direct targets of c-di-GMP, as shown in other bacteria [15], [17], [68], [71]. The *rrp1* mutant did not have an apparent defect in motility, suggesting that c-di-GMP controls other bacterial factor(s) that are important to spirochetal survival in ticks. Note that although c-di-GMP may regulate transcription of flagellar genes [71], our microarray analysis indicates that *flaB* expression is not affected by *rrp1* deletion and thus using the *flaB* as the reference gene in this study remains valid. In addition, expression of previously identified genes important for spirochetal survival in ticks, including *ospA/B, bptA, dps, bb0365* and *lp6.6* [34], [72], [73], [74], [75], [76], were not affected by Rrp1 (**Table 4**). Although glycerol transport/metabolism is important to the optimal growth of *B. burgdorferi* in ticks, independent expression of the glycerol transport/metabolism genes in the *rrp1* mutant does not fully rescue spirochete survival in ticks, and the *rrp1/flaB-glp* spirochetes remain incapable of completing its entire enzootic cycle (**Table 6**). Thus, c-di-GMP likely controls yet-to-be-identified factor(s) that contribute to *B. burgdorferi* proliferation in ticks. In this regard, relatively few c-di-GMP targets have been identified in other bacteria to date. The best characterized c-di-GMP targets are PilZ domain-containing proteins, such as cellulose synthase subunit BcsA in *Gluconacetobacter xylinus* and motility regulatory protein YcgR in *Escherichia coli* [77], [78]. The *B. burgdorferi* genome encodes one PliZ protein, PlzA (BB0733) [79]. Interestingly, Freedman *et al*. showed that *plzA* expression is upregulated during tick feeding, suggesting a potential role of PlzA in the tick vector [79]. Whether PlzA plays a role in the enzootic cycle of *B. burgdorferi* remains to be determined.

Microarray analyses from this study and previous studies by Roger *et al* [32] suggest that expressions of several membrane-associated proteins including Rev, Mlps, and Erps are influenced by Rrp1. Whether these proteins/lipoproteins contribute to *B. burgdorferi* survival in ticks needs to be further determined. In addition, there are some significant differences between these two microarray results. Roger *et al*. showed that Rrp1 influences expression of more than 140 genes, most of which are chromosome-encoded core genes [32]. Our study reveals only few chromosome-encoded genes whose expression was affected by *rrp1* deletion and such effect could be further restored in *rrp1^com^*. One difference between the two studies is the strain used. In this study, an infectious strain B31 5A4NP1 that contains all endogenous plasmids was used, whereas Rogers *et al*., a non-infectious strain B31 5A13 that lost lp25 was used [32]. In addition, differences in media used for cultivation of B. *burgdorferi* might also contribute to differences of the results (we used BSK-II whereas Roger *et al*. used commercially purchased BSK-H complete medium [32]). Another factor that may contribute to this discrepancy is that many genes revealed by WT/*rrp1* microarray analysis could not be confirmed by *rrp1^com^/rrp1* analysis. In fact, there are only 39 genes whose dependence on Rrp1 could be confirmed by *rrp1^com^/rrp1* microarray analysis. We do not fully understand what might contribute to this phenomenon, but it may reflect the complexity of *B. burgdorferi* plasmid contents and gene regulation. Nevertheless, since the expression of *rrp1* as well as the *in vitro* growth defect and the tick survival defect of the *rrp1* mutant were fully restored in *rrp1^com^*, the difference between the microarray results of *WT/rrp1* and *rrp1^com^/rrp1* is not due to Rrp1 and does not affect the overall conclusion of the work presented in the manuscript. The difference of microarray results observed herein also raises caution on microarray analysis of *B. burgdorferi* gene expression and reinforces the importance of performing complementation experiments for identification of genes that are truly affected by inactivation of the target gene.

What signal activates Rrp1 during tick feeding? As a two-component response regulator, the diguanylate cyclase activity of Rrp1 is dependent on phosphorylation [8]. The predicted cognate histidine kinase for Rrp1 is Hk1. Bioinformatics analysis suggests that Hk1 contains a periplasm-located sensor domain homologous to the family 3 periplasmic substrate-binding proteins (SBP_3) [80]. Proteins in this family often bind to amino acids or opine molecules [80], suggesting that *B. burgdorferi* may sense such a molecule and activates the c-di-GMP signaling pathway to achieve successful adaptation of the harsh environments of feeding ticks.

In summary, the findings on Hk1-Rrp1 and Hk2-Rrp2 two-component systems suggest a seemingly simple signal transduction model in *B. burgdorferi*. Through evolution, *B. burgdorferi* reduced its genome and only kept these two sets of two-component systems for the adaptation to each of the two hosts encountered in its entire enzootic life cycle. When spirochetes migrate from ticks to the mammalian host, the Hk2-Rrp2 pathway is activated during tick feeding, leading to the production of OspC, DbpA/B, BBK32 BBA64 and many factors that are important for *B. burgdorferi* to establish infection in the mammalian host [81], [82], [83], [84], [85], [86]. Prior and/or after spirochetes enter the tick gut from mammals, the Hk1-Rrp1 pathway becomes activated, leading to activation of the glycerol pathway and other yet-to-be identified factors to ensure that spirochetes can successfully adapt and replicate in the tick vector.

## Materials and Methods

### Ethics statement

All animal experimentation was carried out in strict accordance with the recommendations in the Guide for the Care and Use of Laboratory Animals of the National Institutes of Health. The protocol of using ticks and mice was approved by the Committee on the Ethics of Animal Experiments and the Institutional Animal Care and Use Committee of Indiana University (Permit Number: 2976). All surgery was performed under sodium pentobarbital anesthesia, and all efforts were made to minimize suffering.

### Bacterial strains and culture conditions

Low–passage, virulent *B. burgdorferi* strain 5A4NP1 (**Table 1**) (a gift from Drs. H. Kawabata and S. Norris, University of Texas Health Science Center at Houston) was derived from wild-type strain B31 by inserting a kanamycin-resistance marker into the restriction modification gene *bbe02* on plasmid lp25 [87]. Borreliae were cultivated in Barbour-Stoenner-Kelly (BSK-II) medium [88] supplemented with 6% normal rabbit serum (Pel Freez Biologicals, Rogers, AR) at 35°C with 5% CO_2_. BSK-glycerol medium was prepared as previously reported by von Lackum and Stevenson, by replacing glucose with an equal amount of glycerol (0.6%) and regular CMRL 1066 with glucose-free CMRL [37]. Relevant antibiotics were added to the cultures with the following final concentrations: 200 µg/ml for kanamycin, 100 µg/ml for streptomycin, 50 µg/ml for gentamicin, and 50 ng/ml for erythromycin. The constructed suicide vectors were maintained in *E. coli* strain TOP10.

### Construction of the *rrp1* mutant and the repaired (*cis*-complementation) strain

The *rrp1* (*bb0419*) mutant was created by allelic exchange in 5A4NP1 by transforming a suicide vector pXY307 (**Fig. 1A**). To construct pXY307, a 2663bp sequence from *B. burgdorferi* chromosome DNA between the coordinates 428794 and 431456 was PCR cloned into pGEM-T (Promega, Madison, WI) using primers pri-Rrp1-40 and pri-Rrp1-41 (**Text S1**). Then an antibiotic marker *flaBp-aadA* was inserted into the Xba*I* site within the *rrp1* gene. The protocol used for transformation was described previously [34], [89]. Numerous streptomycin- and kanamycin-resistant transformants were obtained and the loss of Rrp1 was confirmed by immunoblotting analyses. Endogenous plasmid profiles were determined as previously described [90], [91]. One of the *rrp1* mutant clones that had plasmid profiles identical to the parental strain (5A4NP1) was chosen for further study.

For *cis*-complementation of the *rrp1* mutant, a suicide vector, pMH38, was constructed (**Fig. 1A**). pMH38 contains an *ermC* antibiotic marker flanked by 1) a PCR fragment of *rrp1* and part of the *hk1* (*bb0420*) region (using primers priRrp1-F2-PstI-3 and priRrp1-F2-XhoI-5, **Text S1**) and 2) a PCR fragment of the *bb0418* region (with primers priRrp1-F1-SpeI-5 and priRrp1-F1-BamHI-3). pMH38 DNA was transformed into the *rrp1* mutant. Erythromycin- and kanamycin-resistant transformants were subjected to immunoblot analyses to confirm the restoration of *rrp1* expression. A successfully complimented clone (*rrp1^com^*) with endogenous plasmid profiles identical to the parental strain was then chosen for further study.

### Inactivation and *cis*-complementation of *bb0240-bb0243* (the *glp* operon)

To construct a suicide vector for inactivation of *bb0240-bb0243*, regions of DNA corresponding to 1.9 kb upstream of *bb0240* and 1.9 kb downstream of *bb0240* (including part of *bb0240*) were PCR amplified from B31-A3 genomic DNA. The resulting DNA fragments were then cloned upstream and downstream of a gentamicin-resistant marker (*aacC*) within the pCR-XL-TOPO cloning vector, resulting in suicide vector pMH85R (**Fig. 6A**). The construct was confirmed by sequencing. The plasmid DNA was transformed into *B. burgdorferi* B31 strain 5A4NP1, resulting in a mutant with a disrupted *bb0240* and its promoter region by an *aacC* marker. Since *bb0240-bb0243* constitute an operon, the loss of *bb0240, bb0241, bb0242,* and *bb0243* expression was confirmed by RT-PCR analysis. One of the *bb0240-bb0243* mutant clones (designated as the *glp* mutant) that had all the endogenous plasmids (identical to the parental strain 5A4NP1) was chosen for further study. However, this clone subsequently lost lp28-4 during storage, which may contribute to the reduced infectivity in mice with tick infestation (**Table 5**).

For cis-complementation of the *glp* mutant, the fragment containing the *aacC* marker and the disrupted *bb0240* in pMH85R was replaced with an *aadA* marker linked to a wild-type copy of *bb0240*, *to* generate the suicide vector pMH89 (**Fig. 6A**). pMH89 DNA was then transformed into the *glp* mutant. Restoration of *bb0240-bb0243* expression in the streptomycin/kanamycin-resistant transformants were confirmed by RT-PCR analysis. A positive clone (designated as *glp^com^*) with a plasmid profile identical to the parental strain was selected for further study.

### Construction of the *rrp1* mutant harboring a *bb0240-bb0243* operon (the *glp* operon) driven by the *flaB* promoter

A *flaB* promoter and *bb0240* fusion fragment was constructed using a two-step PCR method. First, a *flaB* promoter fragment was PCR amplified from B31 genomic DNA with primers 240P7Aat2 and 240P8 (**Text S1**). Second, a promoter-less *bb0240* fragment was PCR amplified with primers 240P3B and 240P4. These two overlapping fragments were mixed together and subjected to RCR reaction with 5 cycles. The mixture was then served as template for PCR amplification of the fused *flaBp-bb0240* fragment with primers 240P7Aat2 and 240P4. The *flaBp-bb0240* fragment was cloned into a cloning vector, pSCB-kan/amp, to generate plasmid pMH86. A 1.6 kbp fragment upstream of *bb0240* (starting from 205 bp upstream of the *bb0240* ORF) was PCR amplified with primers 240P9Sal1 and 240P10Aat2. This fragment was then cloned into pMH86 upstream of the *flaB* promoter to generate plasmid pMH87. Lastly, a gentamicin-resistant marker, *aacC*, was inserted into pMH8 upstream of the *flaB* promoter to generate the suicide vector pMH88R (**Fig. 8A**). pMH88R DNA was transformed into the *rrp1* mutant, and gentamicin/streptomycin/kanamycin-resistant clones were selected and subjected to PCR analysis to confirm the replacement of the native *bb0240* with *flaB-bb0240* in the *rrp1* mutant. Constitutive expression of *bb0240-bb0243* in these clones was also determined by quantitative RT-PCR analysis (**Fig. 8B**). Plasmid profiles were then performed, and a clone having a plasmid profile identical to the parental strain was selected for further study. This strain is designated as *rrp1/flpB-glp*.

### Mouse infection via needle inoculation

Four-week-old C3H/HeN mice (Harlan, Indianapolis, IN) were subcutaneously inoculated with 1×10^5^ spirochetes. Ear punch biopsies were collected 14 days after inoculation, and mice were sacrificed by CO_2_ asphyxiation at 21 days post-inoculation. To culture *B. burgdorferi*, ear punch tissue samples were transferred to 2 ml of the BSK-II medium (Sigma-Aldrich, St. Louis, MO) containing an antibiotic mixture of fosfomycin (2 mg/ml), rifampin (5 mg/ml), and amphotericin B (250 µg/ml) (Sigma-Aldrich). All cultures were maintained at 34°C and examined for the presence of spirochetes every 5 to 7 days by dark-field microscopy beginning 5 days after inoculation. A single growth-positive culture was used as the criterion for infection of each mouse.

### Tick-mouse cycle of *B. burgdorferi*

The colony of *Ixodes scapularis* originated from females obtained from Bridgeport, Connecticut, and was maintained in the Tick-Borne Disease Activity Laboratory at the Centers for Disease Control and Prevention, Ft. Collins, Colorado. The tick-mouse experiments were conducted in the Vector-Borne Diseases Laboratory at Indiana University School of Medicine, Indianapolis, IN. Unfed, larvae were fed on groups of mice (C3H/HeN, three mice/group, 100–150 larvae/mouse) that were needle-infected with either 5A4NP1 or various mutant spirochetes. Ticks were allowed to feed to repletion (3–5 days) and then collected within 24 hrs. A portion of fed larvae were subjected to IFA or qPCR analysis (see below). The remaining fed larvae were maintained in the tick incubator and allowed to molt to the nymphal stage (about 5 weeks). One month after molting, unfed nymphs were then allowed to feed on naïve mice (10 ticks per mouse). Fully engorged nymphal ticks were collected within 24 hrs of repletion and subjected to IFA or qPCR analyses. Two weeks after tick feeding, mouse tissues were collected and tested for infection by cultivation for positive growth of spirochetes in BSK-H medium, as described above.

To generating artificially infected ticks with *B. burgdorferi*, a previously described microinjection method was used [26], [34], [35]. Briefly, 0.1 µl of *B. burgdorferi* culture with a concentration of 10^8^ spirochetes per ml was injected into the rectal aperture of unfed nymphal ticks by using a femtojet microinjector system (Eppendorf AG). After microinjection, ticks were placed on naïve C3H/HeN mice (10 ticks per mouse), allowed to feed to repletion (4–5 days), and then collected for IFA or qPCR analysis.

### SDS-PAGE and immunoblot analysis

Spirochetes were harvested by centrifugation at 7,000×*g* and washed three times with PBS (pH 7.4) at 4°C. Pellets were resuspended in SDS buffer containing 50 mM Tris-HCl (pH 8.0), 0.3% sodium dodecyl sulfate (SDS) and 10 mM dithiothreitol (DTT). Total protein lysates (5×10^7^ cells per lane) were separated by 12.5% SDS-polyacrylamide gel electrophoresis (PAGE) and transferred to nitrocellulose membranes (GE-Healthcare, Milwaukee, WI). Protein bands were detected using a 1∶20 dilution of monoclonal antibody against Rrp1 or FlaB, and a 1∶1000 anti-mouse IgG-peroxidase-conjugate secondary antibody (Jackson ImmunoResearch Laboratories, West Grove, PA), followed by development with 4-chloro-1-naphthol as the substrate. Monoclonal antibody against FlaB, 8H3-33, has been described previously [92], [93]. Anti-Rrp1 monoclonal antibody was generated by immunizing BALB/c mice with the full-length fusion protein according to previously published protocols [92].

### Quantitative RT-PCR

RNA samples were extracted from *B. burgdorferi* cultures using the RNeasy mini kit (Qiagen, Valencia, CA) according to the manufacturer's protocols. Three independent culture samples were used for each strain. Digestion of contaminating genomic DNA in the RNA samples was performed using RNase-free DNase I (Promega), and removal of DNA was confirmed by PCR amplification for the *B. burgdorferi flaB* gene. The cDNA was synthesized using the SuperScript III reverse transcriptase with random primers (Invitrogen, Carlsbad, CA). To quantify the transcript levels of interested genes, an absolute quantitation method was used by creating a standard curve in qPCR assay by following the manufacturer's protocol (Strategene, La Jolla, CA). Briefly, a cloning vector containing the *flaB* gene serves as standard template. A series of ten-fold dilution (10^0^ to 10^7^ copies/µl) of the standard template was prepared and qPCR was performed to generate a standard curve by plotting the initial template quantity against the Ct values for the standards. The quantity of the targeted genes and *flaB* in cDNA samples were calculated by comparing their Ct values of the Standard Curve plot. Both standards and samples were performed in triplicate on an ABI 7000 Sequence Detection System using GREEN PCR Master Mix (ABI, Pleasanton, CA). Levels of target gene transcript were reported as per 1000 copies of *flaB* transcripts.

### Indirect immunofluorescence assay (IFA)

IFA was performed as described previously [26]. Briefly, the entire contents of a fed tick were smeared and fixed on a silylated microscope slide (CEL Associates, Pearland, TX). The slides were incubated with BacTrace fluorescein isothiocyanate-conjugated goat anti-*B. burgdorferi* antibody (Kirkegaard and Perry Laboratories Gaithersburg, MD) at 37°C. Samples were observed using an Olympus BX50 fluorescence microscope. Ten ticks from each group were examined by IFA.

### Enumeration of spirochetes in ticks by qPCR

DNA was isolated from engorged larvae (pools of 3 larvae per sample), and replete nymphs (one nymph per sample) using the DNeasy Blood & Tissue Kit B (QIAGEN, CA) according to the manufacturer's instructions. Spirochete burdens within infected ticks were assessed by qPCR with primer pairs of qflaB-F/R for the *B. burgdorferi flaB* gene and qTactin-F/R for the tick actin gene (**Text S1**). Calculations of relative DNA copy number (represented by *flaB*) were normalized with the copy numbers of the tick actin gene.

### Microarray analysis

Wild-type, the *rrp1* mutant and *rrp1^com^* strains were cultivated in BSK-II at 35°C and harvested at the mid-logarithmic growth. RNA was extracted from three biological replicates using Trizol reagent (Invitrogen, Carlsbad, CA) according to the manufacturer's protocol. Digestion of contaminating genomic DNA in the RNA samples was performed using RNase-free DNase I (GenHunter Technology, Nashville, TN), and removal of DNA was confirmed by PCR amplification using primers specific for the *B. burgdorferi flaB* gene. RNA quality was determined using the Agilent Bioanalyzer 2100 (Agilent Technologies, Santa Clara, CA). 70-mer oligonucleotides arrays of *B. burgdorferi* were prepared as previously reported [26], [29], [94]. cDNA synthesis, sample labeling, hybridization, and data analysis were also described previously [26]. A cutoff value of a 3-fold change was used for selecting candidate genes. Statistical analyses were performed using the one and two-sample significance test (p<0.05) in the Acuity program. The array data has been deposited at http://www.ncbi.nlm.nih.gov/geo/ (accession number GSE26968).

### Statistical analysis

To determine the statistical significance of differences observed in qRT-PCR, qPCR, and growth curves, values were compared using an unpaired *t* test. The *P* values are indicated in each figure.

## Supporting Information

Supplemental Text S1 Supplemental Table S1 includes the comparison of the transcriptomes between the wild type and the *rrp1* mutant strains. Supplemental Table S2 includes the comparison of the transcriptomes of the *rrp1^com^* and the *rrp1* mutant spirochetes. Supplemental Table S3 includes sequences of oligonucleotides used in this study.
(DOC)

Supplemental Figure S1 Histopathology of Lyme arthritis in mice infected with B. burgdorferi strains. Four-week-old female C3H/SCID mice were intradermally infected with *B. burgdorferi* strains (1×10^5^ spirochetes per mouse) or with BSK-II medium. Three weeks after inoculation, the rear ankle joint was taken from each mouse and fixed in 10% buffered formalin for more than 48 hours. The specimens were demineralized in a solution of 10% EDTA and 4% PBF phosphate-buffered formalin (7∶3 ratio; two changes) for one week at 4°C with agitation. Following demineralization the specimens were rinsed for two hours with running tap water, and then dehydrated with a series of ethanol solution (70%, 80%, 95%, 100%; 45 minutes per step), cleared in two changes of xylenes (45 minutes each) and infiltrated through 4 changes of melted paraffin (∼60°C; 45 minutes each). The specimens were then embedded in melted paraffin and allowed to harden. Thin 5 µm sections were cut using a rotary microtome equipped with disposable steel knives. Sections were flattened on a heated water bath, floated onto microscope slides and dried. For the H&E (hematoxylin and eosin) staining, the slides were de-paraffinized in xylenes; rehydrated through a graded series of ethanols (70%, 80%, 95%, 100%; 45 minutes per step); stained for 3 minutes in Harris hematoxylin; rinsed in water; de-stained in acid ethanol; rinsed in water; blued the hematoxylin in ammonia water; rinsed in water; counter-stained with eosin (40 seconds), dehydrated, cleared and cover—slipped with a xylenes based mounting media. Original magnification: 10x. Note the inflammatory infiltration in mice infected with wild-type spirochetes (WT), the *rrp1* mutant (*rrp1*), or the complemented spirochetes (*rrp1^com^*), (indicated by arrows), but not in uninfected mice (control).
(TIF)
