# Supplementary material for: Cyclic di-GMP is Essential for the Survival of the Lyme Disease Spirochete in Ticks
Source: PLoS Pathog. 2011 Jun 30;7(6):e1002133. doi: 10.1371/journal.ppat.1002133 (PMC3128128; doi:10.1371/journal.ppat.1002133)
Supplement: Supplemental Text S1 — Supplemental Table S1 includes the comparison of the transcriptomes between the wild type and the rrp1 mutant strains. Supplemental Table S2 includes the comparison of the transcriptomes of the rrp1com and the rrp1 mutant spirochetes. Supplemental Table S3 includes sequences of oligonucleotides used in this study. (DOC) [file ppat.1002133.s001.doc]

**Supplemental Table S1. The comparison of the transcriptomes of the wild type and the *rrp1*** mutant.

| **Locus** | **Common Name** | **Fold change**  **WT/ *rrp1*** | **P value** |
| --- | --- | --- | --- |
|  |  |  |  |
| BB0027 | hypothetical protein | 0.25 | 0.000014 |
| BB0041 | conserved hypothetical protein | 3.301 | 0.000195 |
| BB0240 | glycerol uptake facilitator | 5.534 | 0.000002 |
| BB0241 | glycerol kinase | 7.739 | 0.000001 |
| BB0242 | hypothetical protein | 6.92 | 0.000002 |
| BB0243 | glycerol-3-phosphate dehydrogenase, anaerobic | 6.476 | 0 |
| BB0320 | hypothetical protein | 3.361 | 0.000011 |
| BB0322 | hypothetical protein | 3.326 | 0.000001 |
| BB0369 | ATP-dependent Clp protease, subunit A | 0.312 | 0.000009 |
| BB0406 | hypothetical protein | 0.298 | 0.000039 |
| BB0467 | conserved hypothetical protein | 0.265 | 0.000025 |
| BB0581 | DNA recombinase | 0.319 | 0.000001 |
| BB0590 | dimethyladenosine transferase | 3.226 | 0.000058 |
| BB0692 | hypothetical protein | 0.241 | 0.000003 |
| BB0694 | signal recognition particle protein | 0.309 | 0.000015 |
| BB0771 | RNA polymerase sigma factor | 5.284 | 0.000003 |
| BB0844 | hypothetical protein | 3.788 | 0.000028 |
| BB0848.1 | hypothetical protein, pseudogene | 4.11 | 0.000025 |
| BBA05 | antigen, S1 | 6.589 | 0.000004 |
| BBA06 | hypothetical protein | 9.95 | 0 |
| BBA07 | chpAI protein, putative | 6.697 | 0.000001 |
| BBA22 | hypothetical protein | 0.248 | 0.000021 |
| BBA28 | hypothetical protein | 4.685 | 0.000985 |
| BBA32 | hypothetical protein | 6.194 | 0.000013 |
| BBA33 | hypothetical protein | 5.228 | 0.000185 |
| BBA56 | hypothetical protein | 0.311 | 0.000003 |
| BBA73 | antigen, P35, putative | 3.301 | 0.000346 |
| BBB10 | conserved hypothetical protein | 4.443 | 0.000062 |
| BBB11 | conserved hypothetical protein | 3.313 | 0.000276 |
| BBB13 | conserved hypothetical protein | 3.296 | 0.001149 |
| BBB19 | outer surface protein C | 3.029 | 0.000057 |
| BBD07 | hypothetical protein | 5.142 | 0.00127 |
| BBD19 | hypothetical protein | 3.166 | 0.000024 |
| BBD20 | transposase-like protein, authentic frameshift | 3.049 | 0.000028 |
| BBF001.1 | conserved hypothetical protein, pseudogene | 3.617 | 0.027017 |
| BBF01 | erpD protein, putative | 7.462 | 0.001408 |
| BBF06 | conserved hypothetical protein | 4.041 | 0.027215 |
| BBF10 | hypothetical protein | 0.323 | 0.000032 |
| BBF17 | hypothetical protein | 6.596 | 0.001014 |
| BBF18 | transposase-like protein, authentic frameshift | 5.001 | 0.000019 |
| BBF19 | transposase-like protein, authentic frameshift | 3.651 | 0.000011 |
| BBF22 | protein p23, putative | 3.335 | 0.000406 |
| BBF23 | conserved hypothetical protein | 7.571 | 0 |
| BBF24 | plasmid partition protein, putative | 8.288 | 0 |
| BBF25 | conserved hypothetical protein | 7.356 | 0.000001 |
| BBF26 | conserved hypothetical protein | 11.183 | 0.000007 |
| BBF26.1 | conserved hypothetical protein, pseudogene | 7.993 | 0 |
| BBF27 | hypothetical protein | 9.146 | 0 |
| BBF28 | hypothetical protein | 8.051 | 0.000001 |
| BBG02 | conserved hypothetical protein | 0.252 | 0.000046 |
| BBG10 | hypothetical protein | 0.25 | 0.000042 |
| BBH26 | hypothetical protein | 7.132 | 0.000001 |
| BBH27 | conserved hypothetical protein | 4.636 | 0.000004 |
| BBH41 | conserved hypothetical protein | 4.097 | 0.000125 |
| BBJ01 | hypothetical protein | 3.252 | 0.000001 |
| BBJ11 | hypothetical protein | 0.25 | 0.000457 |
| BBJ12 | hypothetical protein | 0.174 | 0.00231 |
| BBJ12.1 | hypothetical protein, paralogous family 124, pseudogene | 0.129 | 0.002039 |
| BBJ13 | hypothetical protein | 0.11 | 0.000151 |
| BBJ15 | hypothetical protein | 0.17 | 0.000001 |
| BBJ16 | conserved hypothetical protein | 0.297 | 0.000084 |
| BBJ23 | hypothetical protein | 4.353 | 0 |
| BBK41 | hypothetical protein | 0.325 | 0.000053 |
| BBK52.1 | conserved hypothetical protein, pseudogene | 9.048 | 0.000026 |
| BBK53 | outer membrane protein | 3.411 | 0.000016 |
| BBL29 | conserved hypothetical protein | 3.286 | 0.000002 |
| BBL30 | conserved hypothetical protein | 4.135 | 0.002159 |
| BBL31 | conserved hypothetical protein | 4.115 | 0.000103 |
| BBL36 | conserved hypothetical protein | 3.234 | 0.000003 |
| BBL37 | conserved hypothetical protein | 3.12 | 0.000039 |
| BBL39 | erpA protein | 4.141 | 0.000873 |
| BBM27 | rev protein | 19.358 | 0 |
| BBM28 | lipoprotein | 15.086 | 0.000003 |
| BBM29 | conserved hypothetical protein | 13.579 | 0.000013 |
| BBM30 | conserved hypothetical protein | 3.32 | 0.001332 |
| BBM31 | conserved hypothetical protein | 4.237 | 0.004683 |
| BBM32 | plasmid partition protein, putative | 4.182 | 0.000002 |
| BBM33 | conserved hypothetical protein | 5.152 | 0.002165 |
| BBM34 | conserved hypothetical protein | 3.331 | 0.000006 |
| BBM35 | conserved hypothetical protein | 3.115 | 0.000004 |
| BBM36 | conserved hypothetical protein | 4.452 | 0.000238 |
| BBM37 | conserved hypothetical protein | 3.096 | 0.004387 |
| BBM38 | erpK protein | 12.12 | 0.000008 |
| BBM39 | hypothetical protein | 10.309 | 0.000002 |
| BBN11 | hypothetical protein | 3.33 | 0.000235 |
| BBN18 | hypothetical protein, paralogous family 160, authentic point mutation | 3.16 | 0.000187 |
| BBN22 | conserved hypothetical protein, authentic frameshift | 5.352 | 0 |
| BBN26 | outer surface protein, putative | 3.057 | 0.004373 |
| BBN27 | conserved hypothetical protein | 9.733 | 0 |
| BBN28 | lipoprotein | 18.618 | 0.000002 |
| BBN29 | hypothetical protein, paralogous family 161, authentic point mutation | 3.685 | 0.000426 |
| BBN30 | conserved hypothetical protein | 5.118 | 0.00032 |
| BBN31 | conserved hypothetical protein | 10.833 | 0.000005 |
| BBN32 | plasmid partition protein, putative | 4.631 | 0.001976 |
| BBN33 | conserved hypothetical protein | 16.485 | 0 |
| BBN34 | conserved hypothetical protein | 5.464 | 0.001015 |
| BBN36 | conserved hypothetical protein | 4.029 | 0.001015 |
| BBN38 | erpA protein | 14.995 | 0 |
| BBN39 | erpB2 protein | 6.073 | 0.000026 |
| BBN41 | hypothetical protein | 5.354 | 0.000127 |
| BBN42 | hypothetical protein | 3.155 | 0.00001 |
| BBO30 | conserved hypothetical protein | 3.755 | 0.00235 |
| BBO31 | conserved hypothetical protein | 4.418 | 0.00137 |
| BBO32 | plasmid partition protein, putative | 4.162 | 0.000001 |
| BBO37 | conserved hypothetical protein | 3.351 | 0.000187 |
| BBO39 | erpL protein | 12.908 | 0.000002 |
| BBO40 | erpM protein | 4.805 | 0.000189 |
| BBP05 | hypothetical protein | 0.33 | 0.000156 |
| BBP27 | rev protein | 21.461 | 0 |
| BBP28 | lipoprotein | 3.498 | 0.000032 |
| BBP40 | hypothetical protein | 3.587 | 0.003523 |
| BBR32 | conserved hypothetical protein | 3.07 | 0.000018 |
| BBR36 | conserved hypothetical protein | 3.202 | 0.000005 |
| BBR37 | conserved hypothetical protein | 3.121 | 0.002103 |
| BBR40 | erpH protein | 3.228 | 0.000015 |
| BBR41 | conserved hypothetical protein | 3.012 | 0.000009 |
| BBU03 | hypothetical protein | 0.197 | 0.000175 |
| BBU11 | conserved hypothetical protein | 0.019 | 0.000002 |
| BBU12 | conserved hypothetical protein, authentic frameshift | 0.103 | 0.000001 |

|  |
| --- |

**Supplemental Table S2. The comparison of the transcriptomes of *rrp1^com^* and the *rrp1* mutant.**

| **Locus** | **Common Name** | **Fold change *rrp1^com^*/ *rrp1*** | **P value** |
| --- | --- | --- | --- |
| BBM38 | erpK protein | 23.81 | 0.015 |
| BB0040 | chemotaxis protein methyltransferase | 23.26 | 0.022 |
| BBJ02 | hypothetical protein | 18.87 | 0.022 |
| BB0321 | hypothetical protein | 18.52 | 0.021 |
| BB0155 | lipoprotein, putative | 17.86 | 0.020 |
| BBE31 | antigen, P35, putative | 16.39 | 0.014 |
| BBA73 | antigen, P35, putative | 16.13 | 0.003 |
| BB0367 | hypothetical protein | 14.71 | 0.007 |
| BBA65 | hypothetical protein | 14.71 | 0.011 |
| BBA33 | hypothetical protein | 13.33 | 0.036 |
| BBM28 | lipoprotein | 12.82 | 0.001 |
| BBN28 | lipoprotein | 12.35 | 0.003 |
| BBJ23 | hypothetical protein | 12.20 | 0.025 |
| BB0242 | hypothetical protein | 11.36 | 0.015 |
| BB0322 | hypothetical protein | 11.24 | 0.019 |
| BB0212 | hypothetical protein | 10.99 | 0.028 |
| BBC04 | hypothetical protein | 10.99 | 0.048 |
| BB0680 | methyl-accepting chemotaxis protein | 10.53 | 0.003 |
| BBR40 | erpH protein | 10.53 | 0.041 |
| BB0366 | aminopeptidase I | 10.31 | 0.002 |
| BBA64 | antigen, P35 | 10.31 | 0.004 |
| BB0241 | glycerol kinase | 9.35 | 0.007 |
| BBM35 | conserved hypothetical protein | 9.26 | 0.001 |
| BBD24 | hypothetical protein | 8.77 | 0.022 |
| BB0353 | hypothetical protein | 8.40 | 0.028 |
| BB0243 | glycerol-3-phosphate dehydrogenase, anaerobic | 8.26 | 0.005 |
| BB0248 | oligoendopeptidase F | 8.13 | 0.025 |
| BBN35 | conserved hypothetical protein | 8.06 | 0.017 |
| BB0085 | hypothetical protein | 7.94 | 0.010 |
| BB0314 | octaprenyl-diphosphate synthase | 7.75 | 0.021 |
| BBJ01 | hypothetical protein | 7.63 | 0.019 |
| BBA07 | chpAI protein, putative | 7.46 | 0.012 |
| BB0319 | exported protein | 7.35 | 0.043 |
| BB0532 | hypothetical protein | 7.35 | 0.022 |
| BBA01 | conserved hypothetical protein | 7.30 | 0.007 |
| BB0069 | aminopeptidase II | 7.04 | 0.004 |
| BBA71 | hypothetical protein | 6.99 | 0.007 |
| BB0143 | conserved hypothetical protein | 6.80 | 0.025 |
| BB0597 | methyl-accepting chemotaxis protein | 6.76 | 0.024 |
| BBR36 | conserved hypothetical protein | 6.76 | 0.008 |
| BB0249 | phosphatidyltransferase | 6.58 | 0.006 |
| BBU02 | hypothetical protein | 6.49 | 0.007 |
| BBL30 | conserved hypothetical protein | 6.29 | 0.023 |
| BB0136 | penicillin-binding protein | 6.10 | 0.016 |
| BBB09 | hypothetical protein | 6.10 | 0.020 |
| BB0549 | hypothetical protein | 6.02 | 0.045 |
| BBL36 | conserved hypothetical protein | 5.99 | 0.014 |
| BBB11 | conserved hypothetical protein | 5.92 | 0.033 |
| BB0595 | hypothetical protein | 5.88 | 0.017 |
| BB0681 | methyl-accepting chemotaxis protein | 5.88 | 0.003 |
| BB0707 | hypothetical protein | 5.88 | 0.049 |
| BB0070 | conserved hypothetical protein | 5.85 | 0.001 |
| BB0311 | conserved hypothetical protein | 5.78 | 0.015 |
| BB0739 | hypothetical protein | 5.75 | 0.001 |
| BB0305 | hypothetical protein | 5.65 | 0.000 |
| BBO36 | conserved hypothetical protein | 5.65 | 0.016 |
| BBO37 | conserved hypothetical protein | 5.59 | 0.014 |
| BB0063 | hypothetical protein | 5.52 | 0.024 |
| BB0240 | glycerol uptake facilitator | 5.49 | 0.005 |
| BB0728 | NADH oxidase, water-forming | 5.49 | 0.002 |
| BB0564 | hypothetical protein | 5.46 | 0.010 |
| BB0749 | hypothetical protein | 5.46 | 0.042 |
| BBJ26 | ABC transporter, ATP-binding protein | 5.38 | 0.030 |
| BB0151 | N-acetylglucosamine-6-phosphate deacetylase | 5.29 | 0.006 |
| BBA57 | hypothetical protein | 5.26 | 0.032 |
| BB0210 | surface-located membrane protein 1 | 5.18 | 0.008 |
| BBM41 | hypothetical protein | 5.10 | 0.041 |
| BB0207 | UTP--glucose-1-phosphate uridylyltransferase | 5.08 | 0.004 |
| BB0569 | hypothetical protein | 5.05 | 0.016 |
| BB0257 | cell division protein, putative | 5.03 | 0.020 |
| BBB16 | oligopeptide ABC transporter | 5.00 | 0.000 |
| BB0146 | glycine betaine, L-proline ABC transporter | 4.90 | 0.022 |
| BB0385 | basic membrane protein D | 4.76 | 0.050 |
| BB0415 | protein-glutamate methylesterase | 4.72 | 0.020 |
| BB0418 | hypothetical protein | 4.61 | 0.015 |
| BB0627 | vacuolar X-prolyl dipeptidyl aminopeptidase I | 4.59 | 0.004 |
| BB0816 | hypothetical protein | 4.48 | 0.004 |
| BBA26 | hypothetical protein | 4.46 | 0.002 |
| BBA37 | hypothetical protein | 4.46 | 0.035 |
| BBR35 | conserved hypothetical protein, authentic point mutation | 4.46 | 0.001 |
| BBP27 | rev protein | 4.42 | 0.017 |
| BB0660 | GTP-binding protein | 4.41 | 0.021 |
| BB0157 | hypothetical protein | 4.39 | 0.001 |
| BB0328 | oligopeptide ABC transporter | 4.39 | 0.004 |
| BB0598 | UDP-N-acetylmuramate dehydrogenase | 4.39 | 0.018 |
| BB0313 | cell division protein | 4.37 | 0.002 |
| BB0596 | methyl-accepting chemotaxis protein | 4.35 | 0.031 |
| BBM31 | conserved hypothetical protein | 4.33 | 0.030 |
| BB0201 | UDP-N-acetylmuramoylalanyl-D-glutamate--2,6-diaminopimelate ligase | 4.31 | 0.035 |
| BB0437 | chromosomal replication initiator protein | 4.27 | 0.000 |
| BB0454 | lipopolysaccharide biosynthesis-related protein | 4.27 | 0.002 |
| BBE19 | plasmid partition protein, putative | 4.27 | 0.047 |
| BB0689 | hypothetical protein | 4.26 | 0.005 |
| BB0785 | stage V sporulation protein G | 4.22 | 0.002 |
| BB0844 | hypothetical protein | 4.17 | 0.006 |
| BBM27 | rev protein | 4.17 | 0.019 |
| BB0024 | hypothetical protein | 4.13 | 0.017 |
| BB0690 | neutrophil activating protein | 4.12 | 0.004 |
| BBL35 | conserved hypothetical protein | 4.12 | 0.006 |
| BB0349 | hypothetical protein | 4.08 | 0.050 |
| BB0555 | hypothetical protein | 4.03 | 0.011 |
| BB0127 | ribosomal protein S1 | 4.02 | 0.020 |
| BBU05 | plasmid partition protein, putative | 4.00 | 0.019 |
| BBO30 | conserved hypothetical protein | 3.95 | 0.027 |
| BBP28 | lipoprotein | 3.92 | 0.006 |
| BB0068 | conserved hypothetical protein | 3.91 | 0.006 |
| BBA72 | hypothetical protein | 3.91 | 0.009 |
| BB0613 | ATP-dependent protease LA | 3.79 | 0.000 |
| BB0080 | ABC transporter, ATP-binding protein | 3.76 | 0.012 |
| BB0776 | hypothetical protein | 3.76 | 0.010 |
| BB0032 | hypothetical protein | 3.70 | 0.025 |
| BBA24 | decorin binding protein A | 3.65 | 0.013 |
| BB0012 | pseudouridylate synthase I | 3.62 | 0.017 |
| BB0064 | methionyl-tRNA formyltransferase | 3.61 | 0.006 |
| BBB06 | PTS system, cellobiose-specific IIB component | 3.61 | 0.001 |
| BB0536 | zinc protease, putative | 3.60 | 0.009 |
| BBN42 | hypothetical protein | 3.60 | 0.043 |
| BB0261 | hypothetical protein | 3.56 | 0.008 |
| BB0438 | DNA polymerase III, subunit beta | 3.55 | 0.001 |
| BBL39 | erpA protein | 3.53 | 0.025 |
| BBD23 | transposase-like protein, authentic frameshift | 3.52 | 0.010 |
| BB0209 | hypothetical protein | 3.51 | 0.001 |
| BB0058 | hypothetical protein | 3.50 | 0.007 |
| BBL40 | erpB2 protein | 3.48 | 0.009 |
| BBD04 | hypothetical protein, paralogous family 57 | 3.47 | 0.007 |
| BB0308 | hypothetical protein | 3.40 | 0.002 |
| BB0516 | rRNA methylase | 3.40 | 0.040 |
| BB0777 | adenine phosphoribosyltransferase | 3.39 | 0.003 |
| BB0084 | nifS protein | 3.34 | 0.033 |
| BBB29 | PTS system, maltose and glucose-specific IIABC component | 3.33 | 0.002 |
| BB0553 | hypothetical protein | 3.29 | 0.039 |
| BB0662 | hypothetical protein | 3.29 | 0.006 |
| BB0791 | thymidine kinase | 3.28 | 0.019 |
| BBA20 | plasmid partition protein, putative | 3.27 | 0.032 |
| BB0565 | purine-binding chemotaxis protein | 3.26 | 0.011 |
| BB0566 | hypothetical protein | 3.26 | 0.004 |
| BB0770 | conserved hypothetical protein | 3.26 | 0.017 |
| BBO33 | conserved hypothetical protein | 3.26 | 0.042 |
| BB0360 | hypothetical protein | 3.25 | 0.019 |
| BBO31 | conserved hypothetical protein | 3.24 | 0.010 |
| BB0028 | lipoprotein, putative | 3.21 | 0.000 |
| BB0836 | excinuclease ABC, subunit B | 3.18 | 0.027 |
| BB0211 | DNA mismatch repair protein | 3.15 | 0.013 |
| BBL31 | conserved hypothetical protein | 3.14 | 0.006 |
| BB0702 | lipopolysaccharide biosynthesis-related protein | 3.13 | 0.007 |
| BB0245 | hypothetical protein | 3.10 | 0.011 |
| BB0672 | chemotaxis response regulator | 3.10 | 0.001 |
| BBA25 | decorin binding protein B | 3.10 | 0.018 |
| BBB19 | outer surface protein C | 3.09 | 0.038 |
| BBO27 | conserved hypothetical protein | 3.05 | 0.017 |
| BB0253 | ATP-dependent protease LA | 3.04 | 0.001 |
| BB0664 | hypothetical protein | 3.02 | 0.019 |
| BB0467 | conserved hypothetical protein | 0.32 | 0.022 |
| BBR20 | conserved hypothetical protein | 0.32 | 0.022 |
| BBB24 | hypothetical protein | 0.32 | 0.003 |
| BBA45 | conserved hypothetical protein | 0.32 | 0.020 |
| BBG01 | hypothetical protein | 0.32 | 0.010 |
| BBR05 | hypothetical protein | 0.32 | 0.008 |
| BBP23 | pore-forming hemolysin | 0.31 | 0.002 |
| BBA44 | hypothetical protein | 0.31 | 0.009 |
| BBA38 | hypothetical protein | 0.31 | 0.020 |
| BBP05 | hypothetical protein | 0.31 | 0.005 |
| BBO09 | conserved hypothetical protein | 0.30 | 0.000 |
| BBJ44 | hypothetical protein | 0.30 | 0.001 |
| BBM21 | conserved hypothetical protein | 0.30 | 0.010 |
| BB0733 | hypothetical protein | 0.30 | 0.036 |
| BBD18 | hypothetical protein | 0.30 | 0.000 |
| BBK41 | hypothetical protein | 0.30 | 0.019 |
| BBJ15 | hypothetical protein | 0.30 | 0.005 |
| BBR07 | conserved hypothetical protein | 0.30 | 0.004 |
| BBA54 | hypothetical protein | 0.29 | 0.029 |
| BBN20 | conserved hypothetical protein | 0.29 | 0.013 |
| BBA23 | conserved hypothetical protein | 0.29 | 0.028 |
| BBM23 | pore-forming hemolysin | 0.28 | 0.000 |
| BBR23 | pore-forming hemolysin | 0.28 | 0.002 |
| BBJ08 | hypothetical protein | 0.28 | 0.038 |
| BBM06 | conserved hypothetical protein | 0.27 | 0.001 |
| BBB23 | conserved hypothetical protein | 0.27 | 0.023 |
| BBA49 | hypothetical protein | 0.27 | 0.037 |
| BBM12 | hypothetical protein | 0.26 | 0.022 |
| BBA53 | hypothetical protein | 0.26 | 0.044 |
| BBE15 | hypothetical protein | 0.26 | 0.001 |
| BBN10 | conserved hypothetical protein | 0.26 | 0.016 |
| BBO02 | hypothetical protein | 0.26 | 0.001 |
| BB0214 | translation elongation factor P | 0.26 | 0.032 |
| BBA51 | hypothetical protein | 0.26 | 0.027 |
| BBR06 | conserved hypothetical protein | 0.26 | 0.000 |
| BBL19 | conserved hypothetical protein | 0.25 | 0.001 |
| BBM26 | conserved hypothetical protein | 0.25 | 0.028 |
| BBP21 | conserved hypothetical protein | 0.25 | 0.004 |
| BBA61 | conserved hypothetical protein | 0.25 | 0.004 |
| BBM24 | hemolysin accessory protein | 0.25 | 0.001 |
| BBN05 | hypothetical protein, paralogous family 148, authentic frameshift | 0.25 | 0.004 |
| BBA42 | conserved hypothetical protein | 0.25 | 0.012 |
| BBR12 | hypothetical protein | 0.24 | 0.003 |
| BBM15 | hypothetical protein | 0.24 | 0.002 |
| BBL09 | conserved hypothetical protein | 0.24 | 0.001 |
| BBD25 | hypothetical protein | 0.24 | 0.039 |
| BBA46 | hypothetical protein | 0.24 | 0.019 |
| BBR21 | conserved hypothetical protein | 0.24 | 0.009 |
| BBN14 | hypothetical protein | 0.24 | 0.008 |
| BBP09 | conserved hypothetical protein | 0.24 | 0.001 |
| BBM03 | hypothetical protein | 0.24 | 0.015 |
| BBP10 | conserved hypothetical protein | 0.24 | 0.006 |
| BBN04 | hypothetical protein | 0.23 | 0.033 |
| BBN07 | conserved hypothetical protein | 0.23 | 0.030 |
| BBP20 | conserved hypothetical protein | 0.23 | 0.002 |
| BBL12 | hypothetical protein | 0.23 | 0.003 |
| BBL21 | conserved hypothetical protein | 0.23 | 0.007 |
| BBL05 | hypothetical protein | 0.23 | 0.012 |
| BBO10 | conserved hypothetical protein | 0.23 | 0.002 |
| BBH18 | hypothetical protein | 0.23 | 0.001 |
| BBR14 | hypothetical protein | 0.22 | 0.004 |
| BBO21 | conserved hypothetical protein | 0.22 | 0.004 |
| BBC12 | conserved hypothetical protein | 0.22 | 0.006 |
| BBL15 | hypothetical protein | 0.22 | 0.000 |
| BBP12 | hypothetical protein | 0.22 | 0.011 |
| BBM20 | conserved hypothetical protein | 0.22 | 0.001 |
| BBL20 | conserved hypothetical protein | 0.21 | 0.003 |
| BBM11 | hypothetical protein | 0.21 | 0.008 |
| BBN02 | hypothetical protein | 0.21 | 0.002 |
| BBO05 | hypothetical protein | 0.21 | 0.002 |
| BBA40 | hypothetical protein | 0.21 | 0.012 |
| BBG19 | hypothetical protein | 0.21 | 0.028 |
| BBK24.1 | hypothetical protein | 0.21 | 0.005 |
| BBP06 | conserved hypothetical protein | 0.21 | 0.002 |
| BBR04 | hypothetical protein | 0.21 | 0.003 |
| BBL14 | hypothetical protein | 0.20 | 0.002 |
| BBE08 | hypothetical protein | 0.20 | 0.004 |
| BBA22 | hypothetical protein | 0.20 | 0.004 |
| BBO12 | hypothetical protein | 0.20 | 0.004 |
| BBR09 | conserved hypothetical protein | 0.20 | 0.001 |
| BBP14 | hypothetical protein | 0.20 | 0.002 |
| BBP15 | hypothetical protein | 0.19 | 0.004 |
| BBP26 | conserved hypothetical protein | 0.19 | 0.012 |
| BBL11 | hypothetical protein | 0.19 | 0.009 |
| BBE26 | hypothetical protein | 0.19 | 0.011 |
| BBR10 | conserved hypothetical protein | 0.19 | 0.000 |
| BBA50 | hypothetical protein | 0.18 | 0.045 |
| BBN06 | hypothetical protein, paralogous family 149, authentic frameshift | 0.18 | 0.002 |
| BBG17 | hypothetical protein | 0.17 | 0.003 |
| BBA39 | hypothetical protein | 0.17 | 0.020 |
| BBP03 | hypothetical protein | 0.17 | 0.002 |
| BBA41 | conserved hypothetical protein | 0.17 | 0.011 |
| BBN21 | hypothetical protein, paralogous family 141, authentic frameshift | 0.17 | 0.003 |
| BBR02 | hypothetical protein, paralogous family 147, authentic frameshift | 0.17 | 0.004 |
| BBM07 | conserved hypothetical protein | 0.17 | 0.009 |
| BBG18 | hypothetical protein | 0.17 | 0.005 |
| BBL03 | hypothetical protein | 0.17 | 0.001 |
| BBJ41 | antigen, P35, putative | 0.16 | 0.001 |
| BBL04 | hypothetical protein | 0.16 | 0.002 |
| BBL07 | conserved hypothetical protein | 0.16 | 0.007 |
| BBO03 | hypothetical protein | 0.15 | 0.001 |
| BBP07 | conserved hypothetical protein | 0.15 | 0.008 |
| BBK54 | conserved hypothetical protein | 0.15 | 0.002 |
| BBG21 | hypothetical protein | 0.15 | 0.006 |
| BBG33 | conserved hypothetical protein | 0.14 | 0.012 |
| BBD12 | hypothetical protein | 0.14 | 0.018 |
| BBP11 | hypothetical protein | 0.14 | 0.002 |
| BBN09 | conserved hypothetical protein | 0.14 | 0.011 |
| BBG13 | hypothetical protein | 0.14 | 0.000 |
| BBP04 | hypothetical protein | 0.14 | 0.001 |
| BBU09 | conserved hypothetical protein | 0.14 | 0.002 |
| BBU03 | hypothetical protein | 0.14 | 0.008 |
| BBJ09 | outer surface protein D | 0.13 | 0.013 |
| BBA56 | hypothetical protein | 0.13 | 0.019 |
| BBP08 | conserved hypothetical protein | 0.13 | 0.005 |
| BBA55 | hypothetical protein | 0.12 | 0.023 |
| BBO04 | hypothetical protein | 0.12 | 0.001 |
| BBG03 | conserved hypothetical protein, authentic frameshift | 0.12 | 0.001 |
| BBE09 | protein p23 | 0.11 | 0.007 |
| BBO07 | conserved hypothetical protein | 0.11 | 0.002 |
| BBG12 | hypothetical protein | 0.11 | 0.003 |
| BBL08 | conserved hypothetical protein | 0.11 | 0.001 |
| BBN08 | conserved hypothetical protein | 0.10 | 0.002 |
| BBG11 | hypothetical protein | 0.09 | 0.005 |
| BBJ39.1 | multidrug-efflux transporter, pseudogene | 0.09 | 0.003 |
| BBJ39 | hypothetical protein | 0.08 | 0.008 |
| BB0509 | hypothetical protein | 0.08 | 0.004 |
| BBL06 | conserved hypothetical protein | 0.08 | 0.001 |
| BBJ10 | hypothetical protein | 0.04 | 0.023 |
| BBF23 | conserved hypothetical protein | 0.04 | 0.012 |

|  |
| --- |

**Supplemental Table 3**. Sequences of oligonucleotides used in this study

| **Name** | **sequence** | **purpose** |
| --- | --- | --- |
|  |  |  |
| priRrp1-F2-XhoI-5 | CACTCGAGGTTTAACCCAGAAATGGATTTTGAGG | Complement *rrp1* |
| priRrp1-F2-ClaI-3 | CAATCGATCTATTAAATATAAAATTTAATATCTAAACTGATTTC | Complement *rrp1* |
| priRrp1-F1-SpeI-5 | CAACTAGTGTATTTATAAGTTATAGACATTCCAATAGAATCG | Complement *rrp1* |
| priRrp1-F1-BamHI-3 | CAGGATCCCTACGGCTCTTGGGCAAG | Complement *rrp1* |
| priRrp1-40 | ATGGAAGCGGCTTTTCACAAAGCTTTAAAC | inactive *rrp1* |
| priRrp1-41 | TGGATTTTCGGTGGCTAAGGAAATTAG | inactivate *rrp1* |
| 240P3CAat2 | CAGACGTCGAATAATGGCAACTTTCCCTGCTG | Construct the *glp* mutant |
| 240P12Pst1 | CTCTGCAGTTTATGAACTATGAGAATTCTGTATCCAG | Construct the *glp* mutant |
| 240P11Aat2 | CAGACGTCATATTAAATATAATTTTAATAAGGC | Complement the *glp* mutant |
| 240P3B | CATGGAGGAATGACATATGAATTATACAAAATTCCAAG | Construct *flaBp-glp* |
| 240P4 | GTCTTTGTTATTTTTTAGTGTAAATTCGTAAATTGTAGC | Construct *flaBp-glp* |
| 240P7Aat2 | CAGACGTCGATCCAAGATAGAGAGAG | Construct *flaBp-glp* |
| 240P8 | CTTGGAATTTTGTATAATTCATATGTCATTCCTCCATG | Construct *flaBp-glp* |
| 240P9Sal1 | GAGTCGACTTTCAATTTATGGAAGACAAAAACC | Construct *flaBp-glp* |
| 240P10Aat2 | CAGACGTCCTTATTTTTAATTAAGATTAATG | Construct *flaBp-glp* |
| q240F | ACACAGCGACAATCCATTTA | qRT-PCR for *bb0240* |
| q240R | AGCATAACCGTTCATTCCTC | qRT-PCR for *bb0240* |
| q241F | TGAAATTGACGCTATTGGAA | qRT-PCR for *bb0241* |
| q241R | CATTGTAGATGGGCTTTCCT | qRT-PCR for *bb0241* |
| q242F | ATGTTTGGCAAGCAAATAAA | qRT-PCR for *bb0242* |
| q242R | TTGCTCCAATGAATTTTACCT | qRT-PCR for *bb0242* |
| q243F | AATGGATCCAAAAGCTCAAG | qRT-PCR for *bb0243* |
| q243R | TCTATGGCCTTAAGCAAGGT | qRT-PCR for *bb0243* |
| primer A | GAGCAAGCCCAATTGAAACAG | PCR analysis used in Fig. 1B |
| primer B | GATGATTCTCCCCACAATTTAG | PCR analysis used in Fig. 1B |
| primer C | CAGTAGACATTATTTGCCGACTACC | PCR analysis used in Fig. 1B |
| primer D | AGATCTCTTATTAAATAATTTATAGCTATTGAAAAG | PCR analysis used in Fig. 1B |
