# Supplementary figures and images for: Cyclic di-GMP is Essential for the Survival of the Lyme Disease Spirochete in Ticks
Source: PLoS Pathog. 2011 Jun 30;7(6):e1002133. doi: 10.1371/journal.ppat.1002133 (PMC3128128; doi:10.1371/journal.ppat.1002133)

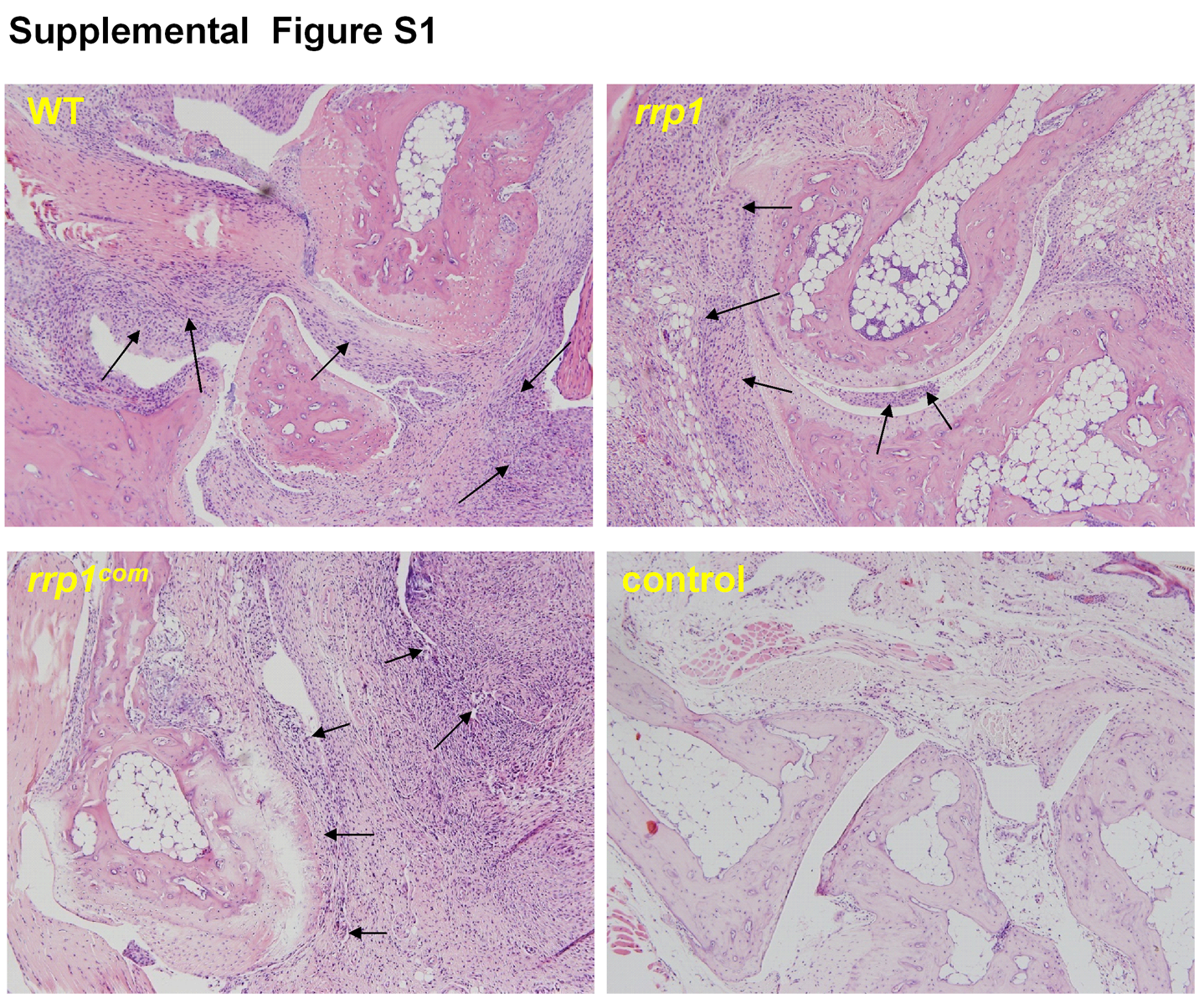

Supplement: Supplemental Figure S1 — Histopathology of Lyme arthritis in mice infected with B. burgdorferi strains. Four-week-old female C3H/SCID mice were intradermally infected with B. burgdorferi strains (1×10^5^ spirochetes per mouse) or with BSK-II medium. Three weeks after inoculation, the rear ankle joint was taken from each mouse and fixed in 10% buffered formalin for more than 48 hours. The specimens were demineralized in a solution of 10% EDTA and 4% PBF phosphate-buffered formalin (7∶3 ratio; two changes) for one week at 4°C with agitation. Following demineralization the specimens were rinsed for two hours with running tap water, and then dehydrated with a series of ethanol solution (70%, 80%, 95%, 100%; 45 minutes per step), cleared in two changes of xylenes (45 minutes each) and infiltrated through 4 changes of melted paraffin (∼60°C; 45 minutes each). The specimens were then embedded in melted paraffin and allowed to harden. Thin 5 µm sections were cut using a rotary microtome equipped with disposable steel knives. Sections were flattened on a heated water bath, floated onto microscope slides and dried. For the H&E (hematoxylin and eosin) staining, the slides were de-paraffinized in xylenes; rehydrated through a graded series of ethanols (70%, 80%, 95%, 100%; 45 minutes per step); stained for 3 minutes in Harris hematoxylin; rinsed in water; de-stained in acid ethanol; rinsed in water; blued the hematoxylin in ammonia water; rinsed in water; counter-stained with eosin (40 seconds), dehydrated, cleared and cover—slipped with a xylenes based mounting media. Original magnification: 10x. Note the inflammatory infiltration in mice infected with wild-type spirochetes (WT), the rrp1 mutant (rrp1), or the complemented spirochetes (rrp1com), (indicated by arrows), but not in uninfected mice (control). (TIF) [file ppat.1002133.s002.tif]
